# Nuclear receptor 4A1 (NR4A1) silencing protects hepatocyte against hypoxia-reperfusion injury *in vitro* by activating liver kinase B1 (LKB1)/AMP-activated protein kinase (AMPK) signaling

**DOI:** 10.1080/21655979.2022.2053804

**Published:** 2022-03-21

**Authors:** Yu Zheng, Yingying Tao, Xiaobo Zhan, Qi Wu

**Affiliations:** a Hepatobiliary Pancreatic Surgery Department, Tongde Hospital of Zhejiang Province, Hangzhou, China; b Emergency Intensive Care Unit, Hangzhou Ninth People’s Hospital, Hangzhou, China

**Keywords:** NR4A1, LKB1/AMPK, ischemia-reperfusion injury, oxidative stress, inflammation, apoptosis

## Abstract

The nuclear receptor 4A1 (NR4A1) is widely involved in the regulation of cell survival and is related to ischemic injury in several organs. This research examined the emerging role and mechanism of NR4A1 in hepatocyte ischemia-reperfusion injury (IRI). BRL-3A cells were subjected to hypoxia-reperfusion (H/R) to simulate an IRI model *in vitro*. The expression of NR4A1 and liver kinase B1 (LKB1)/AMP-activated protein kinase (AMPK) pathway-related proteins (LKB1, AMPK, and ACC) was detected by western blotting or RT-qPCR under H/R condition after NR4A1 overexpression or silencing. Then, radicicol, an inhibitor of LKB1 pathway, was used to determine the role of NR4A1 in hepatocyte H/R injury by regulating LKB1. Under the help of CCK-8 assay, cell viability was assessed. The levels of ROS, MDA, and SOD were determined with corresponding kits to evaluate oxidative stress. Additionally, RT-qPCR was employed to analyze the releases of the inflammatory factors. Flow cytometry was applied to estimate the apoptosis and its related proteins, and autophagy-associated proteins were assayed by western blotting. Results indicated that NR4A1 was highly expressed, while proteins in LKB1/AMPK signaling was downregulated in BRL-3A cells exposed to H/R. The activation of LKB1/AMPK pathway could be negatively regulated by NR4A1. Moreover, NR4A1 depletion conspicuously promoted cell viability, inhibited oxidative stress as well as inflammation, and induced apoptosis and autophagy in H/R-stimulated BRL-3A cells, which were reversed after radicicol intervention. Collectively, NR4A1/LKB1/AMPK axis is a new protective pathway involved in hepatocyte IRI, shedding new insights into the improvement of hepatocyte IRI.

## Graphical abstract

**Figure:**
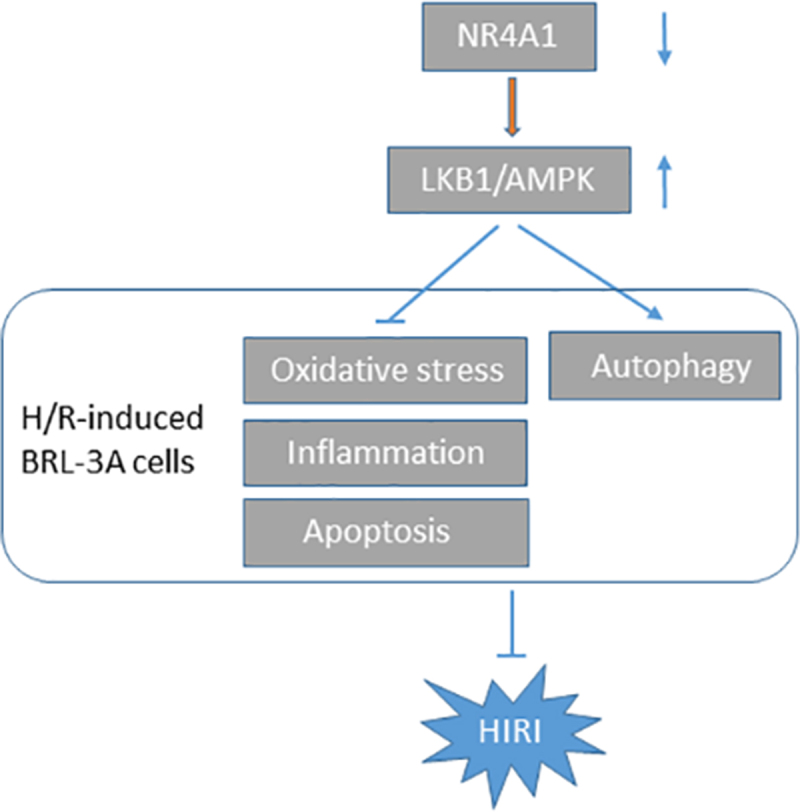

## Introduction

Ischemia-reperfusion injury (IRI) refers to more severe injury of organs after experiencing ischemic injury and resuming blood supply [1]. Among these, hepatic ischemia-reperfusion injury (HIRI) is a common tissue and organ damage in liver surgery and also an important factor affecting the success of liver transplantation [2]. HIRI manifests as impaired liver function in the early stage and hepatic fibrosis or liver failure in the late stage, which greatly reduces the quality of life in patients with liver injury [3]. The pathogenesis of HIRI may be related to the mitochondrial dysfunction, abundant content of reactive oxygen species (ROS), activation of apoptotic kinase, and other factors [4]. Nevertheless, the specific complex mechanism of HIRI remains elusive.

Nuclear receptor 4A1 (NR4A1), one of the orphan nuclear receptors, is widely expressed in various tissues and organs and participates in cellular inflammation, proliferative ability, differentiation, apoptosis, autophagy, and metabolism through transcriptional and non-transcriptional regulation [5]. Data from several studies suggest the critical role of NR4A1 in ischemic injury of organs. For instance, in cerebral ischemic injury, NR4A1 could promote the cerebral ischemic injury through regulating nuclear factor-κB (NF-κB) pathway [6]. In renal ischemic injury, NR4A1 silencing significantly improved the renal IRI in mice by activating β-catenin [7]. Importantly, emerging evidence supports that NR4A1 was highly expressed in the liver tissue of mice after IRI [8]. This implies that NR4A1 may also be involved in HIRI. Nonetheless, the specific role of NR4A1 as well as its mechanism in HIRI haven’t been elucidated till now.

Liver kinase B1 (LKB1) is a ubiquitously expressed and evolutionarily conserved serine threonine kinase that can positively regulate AMP-activated protein kinase (AMPK) [9]. Like NR4A1, LKB1/AMPK axis also exerts a crucial role in IRI diseases. For example, paeoniflorin could ameliorate intestinal IRI and restore autophagy through activating LKB1/AMPK signaling [10]. Sestrin2 could protect heart IRI through the activation of LKB1/AMPK axis [11]. A recent study has reported that NR4A1 interacts with LKB1 through the ligand-binding domain and sequesters it in the nucleus, leading to impeded AMPK activation in hepatocytes [12]. In view of this, we hypothesized that NR4A1 may has a pivotal role in the improvement of hepatic IRI in conjunction with LKB1/AMPK binding.

In this study, hypoxia/reoxygenation (H/R)-induced rat normal liver BRL-3A cells were used to establish a HIRI model *in vitro*. The effects of NR4A1 on oxidative stress, apoptosis, and inflammation as well as the potential mechanisms related to LKB1/AMPK signaling of BRL-3A cells exposed to H/R condition were investigated. Our findings might offer a novel and promising target for the treatment of HIRI.

## Materials and methods

### Cell culture and treatment

Cell Bank of the Type Culture Collection of the Chinese Academy of Sciences (Shanghai, China) was the supplier of rat normal hepatocytes (BRL-3A). Dulbecco’s Modified Eagle Medium (DMEM; Hyclone, Logan, UT, USA) decorating with 10% fetal bovine serum (FBS) was applied to cultivate the cells. BRL-3A cells in the logarithmic phase were harvested for follow-up experimental study. Experiments to induce H/R cell model was carried out as mentioned in previous study [13]. In briefly, the collected cells were cultivated in serum-free DMEM medium and maintained in a 5% CO_2_, and 95% N_2_ hypoxia incubator at 37°C. Cells were cultured in hypoxic conditions for 12 h and then switched to normal medium for another 4 h. Radicicol (Cat no. A4067) was provided by ApexBio (USA). Radicicol (0.05 μM) was used as the LKB1 inhibitor and added into the cultured cells with H/R pretreatment for 5 min.

### Cell transfection

The overexpressed NR4A1 plasmid (Ov-NR4A1) and the corresponding pcDNA3.1 empty vector (Ov-NC) were offered by GenePharma (GenePharma Co., Ltd., Shanghai, China). Small interference RNAs (siRNAs) carrying NR4A1 (siRNA-NR4A1-1 and siRNA-NR4A1-2) and its negative control (siRNA-NC) were purchased from RiboBio. BRL-3A cells that inoculated into 6-well plates (2 × 10^5^ cells/well) were then transfected with above vectors with the application of Lipofectamine 2000 transfection reagent (Invitrogen) in light of the standard procedures of the supplier. The transfection efficiency was detected by reverse transcription-quantitative PCR (RT-qPCR) analysis after 48 h.

### Cell viability assay

Inoculated into 96-well plates at the density of 2 × 10^4^ cells/well for incubation, BRL-3A cells were then added with 10 μl of Cell counting kit (CCK)-8 solution (Beyotime). After 2 h, the absorbance value of each hole was decided by a microplate reader (BioTek microplate reader) in the premise of λ = 450 nm.

### Intracellular reactive oxygen species (ROS) level assay

Fluorescence probe 2’,7’-dichlorofluorescin diacetate (DCFH-DA) was employed to evaluate ROS level. In short, 10 μΜ DCFH-DA (Sigma-Aldrich) was used to treat BRL-3A cells at 37°C for half an hour in the dark and then washed with serum-free DMEM for three times to fully remove the DCFH-DA that has not entered the cells. Finally, a fluorescence spectrometer (HTS7000, Perkin Elmer, USA) was applied to track ROS staining.

### Test for malondialdehyde (MDA) and superoxide dismutase (SOD)

With the adoption of MDA (Cat. no. A003-1-2) and SOD (Cat. no. A001-3-2) assay kits which were procured from Jiancheng Bioengineering Institute (Nanjing, China), the content of MDA and the activity of SOD in collected cell supernatants were assessed in line with the instructions of the kit vendor.

### Determination of inflammatory factors levels

The medium were obtained and centrifuged at 4°C at 850 × g for 10 min to collect the culture supernatant. Enzyme-linked immunosorbent assay (ELISA) kits provided by Beyotime were used to determine the levels of tumor necrosis factor (TNF)-α, interleukin (IL)-1β, IL-6, and monocyte chemoattractant protein-1 (MCP-1) in the supernatant according to the manufacturer’s protocols.

### Flow cytometry

According to the instructions of Annexin V-FITC/PI Apoptosis kit (Sangon, China). 1.25 μl AnnexinV-FITC and 10 μl propidium iodide working solution was added to each group of cell suspension and incubated for 15 min at room temperature. Subsequently, 500 μl of 1 × Annexin binding buffer was added, and FLEX flow cytometry system was utilized to track the apoptotic cells.

### RT-qPCR analysis

The synthetization of total RNA extracted by Trizol reagent (Invitrogen) into complementary DNA (cDNA) was carried out by a reverse transcription kit (Promega). The operation of PCR reaction was conducted by QuantiTect SYBR Green PCR Kits (Qiagen, Inc.) on an ABI PRISM 7300 sequence Detection system (Applied Biosystems, Foster City, CA, USA). Data were analyzed with the help of 2^−ΔΔCt^ method [14]. All values were normalized to Glyceraldehyde-phosphate dehydrogenase (GAPDH). The primer sequences were listed as below: NR4A1, forward, 5’-CCTGGTGTAAGCT-3’, reverse, 5’-GCCTTGGCCAACCACATTAT-3’; GAPDH, forward, 5’-TGTGAACGGATTTGGCCGTA-3’, reverse, 5’-GATGGTGATGGGTTTCCCGT-3’.

### Western blot analysis

RIPA buffer (Beyotime) was applied to isolate total proteins from sample cells, followed by a determination of protein concentration utilizing bicinchoninic acid (BCA) kit (Beyotime). After the separation of protein samples with 10% sodium dodecyl sulfate-polyacrylamide gel electrophoresis (SDS-PAGE), the transferring onto polyvinylidene fluoride (PVDF) membranes was immediately carried out. Prior to the overnight incubation with 1:1000 primary antibodies against NR4A1 (25,851-1-AP, Proteintech, Chicago, IL, USA), phospho (p)-LKB1 (3482S, Cell Signaling Technology, Boston, MA, USA), LKB1 (3047S, Cell Signaling Technology, Boston, MA, USA), p-AMPK (2535 T, Cell Signaling Technology, Boston, MA, USA), AMPK (5832 T, Cell Signaling Technology, Boston, MA, USA), p-acetyl-CoA carboxylase (p-ACC, ab68191, Abcam, Cambridge, UK), ACC (ab68191, Abcam, Cambridge, UK), B-cell lymphoma 2 (Bcl-2, ab196495, Abcam, Cambridge, UK), Bcl-2-associated X protein (Bax, 2772 T, Cell Signaling Technology, Boston, MA, USA), cleaved caspase-3 (9664 T, Cell Signaling Technology, Boston, MA, USA), caspase-3 (9662S, Cell Signaling Technology, Boston, MA, USA), Poly-ADP-Ribose polymerase (PARP, ab191217, Abcam, Cambridge, UK), cleaved PARP (94885S, Cell Signaling Technology, Boston, MA, USA), Beclin1 (3495 T, Cell Signaling Technology, Boston, MA, USA), autophagy-related protein 5 (ATG5, 12,994 T, Cell Signaling Technology, Boston, MA, USA), ATG7 (8558 T, Cell Signaling Technology, Boston, MA, USA), p62 (39749S, Cell Signaling Technology, Boston, MA, USA), and GAPDH (5174 T, Cell Signaling Technology, Boston, MA, USA) at 4°C, 5% nonfat milk was adopted to impede these membranes for 1 h. After that, secondary antibody conjugated to horseradish peroxidase-conjugated (HRP) were utilized to cultivate the membranes at room temperature for another 2 h. Immune bands were detected by enhanced chemiluminescence (ECL) chromogenic kit (Thermo Fisher Scientific, Inc.). The blots were captured with the aid of Image Pro Plus software (Media Cybernetics, Rockville, MD, USA).

### Statistical analysis

GraphPad Prism version 8.0 (GraphPad Software, Inc.) was adopted to analyze the collected data, which were demonstrated as the mean ± standard deviation (SD). Comparisons between two groups were shown by student t-test and differences among multiple groups were compared by one-way analysis of variance (ANOVA) and Tukey’s post hoc test. P < 0.05 was supposed to exhibit statistical difference.

## Results

### NR4A1 is significantly upregulated while proteins in LKB1/AMPK signaling is downregulated in H/R-treated BRL-3A cells

**Figure 1.**
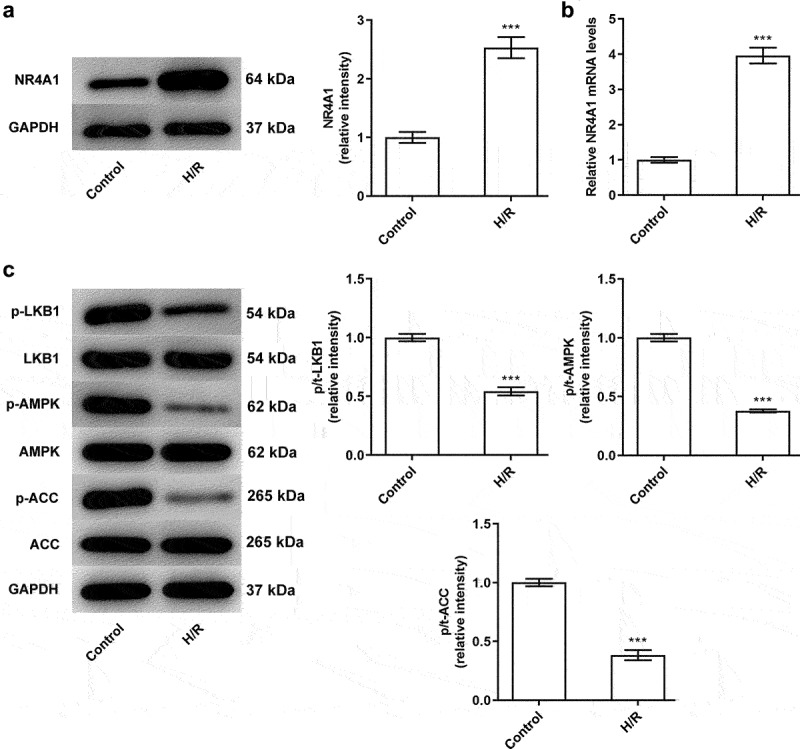
NR4A1 is significantly upregulated while proteins in LKB1/AMPK signaling is downregulated in H/R-treated BRL-3A cells. (a-b) Expression of NR4A1 in BRL-3A cells treated with H/R was detected using western blotting and RT-qPCR. (c) The expression or proteins in LKB1/AMPK signaling in BRL-3A cells treated with H/R were detected by western blotting. ***P < 0.001 vs. control.

To detect the role of NR4A1 in H/R-induced BRL-3A cells, western blotting as well as RT-qPCR was applied to assess the level of NR4A1. As Figure 1a and 1b depicted, NR4A1 expression in H/R group was markedly elevated when compared to the control group. In addition, in contrast with the control group, the phosphorylation levels of LKB1, AMPK, and ACC were conspicuously reduced in H/R group (Figure 1c). Taken together, these results indicated that NR4A1 was upregulated while the phosphorylation levels of LKB1/AMPK pathway-related proteins were downregulated in H/R-treated BRL-3A cells.

### NR4A1 inhibits LKB1/AMPK pathway in BRL-3A cells exposed to H/R

**Figure 2.**
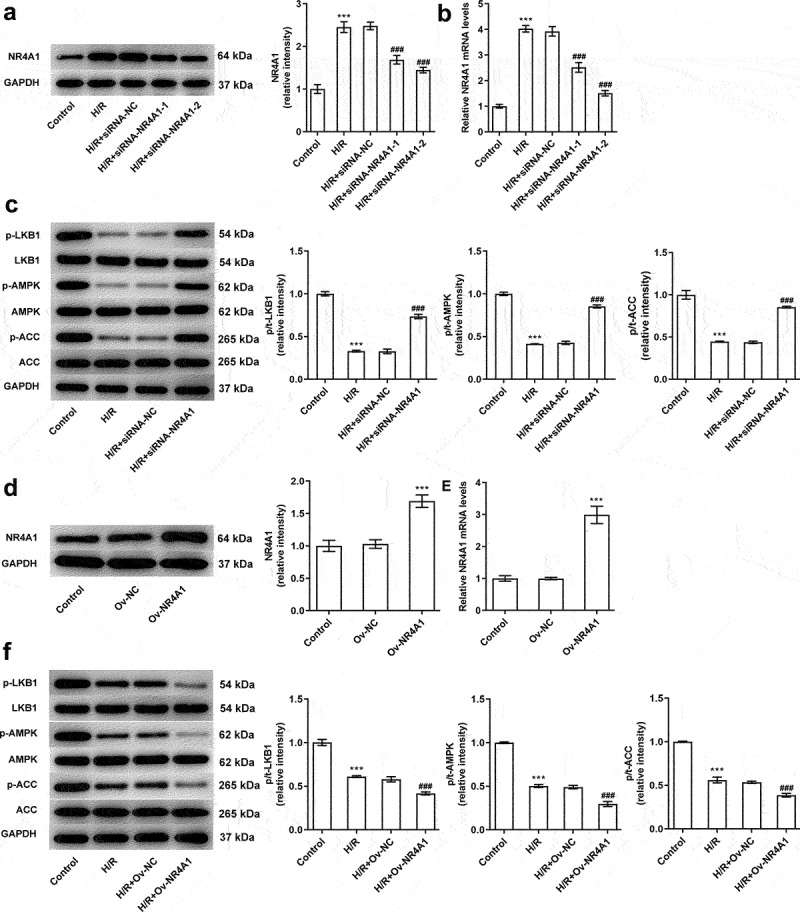
NR4A1 inhibits the activation of LKB1/AMPK pathway in BRL-3A cells treated with H/R. (a-b) siRNA NR4A1-mediated transfection efficiency was determined by western blotting and RT-qPCR. (c) Effect of NR4A1 knockdown on the phosphorylation levels of the LKB1/AMPK signaling in BRL-3A cells treated with H/R were detected by western blotting. ***P < 0.001 vs. control; ###P < 0.001 vs. H/R+ siRNA-NC. (d-e) Western blot and RT-qPCR were used for detecting the transfection efficiency of NR4A1 overexpressed plasmid. ***P < 0.001 vs. ov-NC. (f). Effect of NR4A1 overexpression on the phosphorylation levels of the LKB1/AMPK signaling pathway in BRL-3A cells treated with H/R were detected by western blotting. ***P < 0.001 vs. control; ^###^P < 0.001 vs. H/R+ Ov-NC.

Subsequently, NR4A1 overexpression plasmid or NR4A1 siRNA (siRNA-NR4A1-1 and siRNA-NR4A1-2) were transfected into BRL-3A cells to overexpress or silence NR4A1 expression. In contrast with H/R+ siRNA-NC group, NR4A1 protein and mRNA levels were notably reduced in the H/R+ siRNA-NR4A1-1 and H/R+ siRNA-NR4A1-2 groups (Figure 2a and 2b). The lower NR4A1 expression was observed in the H/R+ siRNA-NR4A1-2 group; therefore, siRNA-NR4A1-2 was selected in the follow-up experiments. Additionally, as exhibited in Figure 1c, NR4A1 knockdown significantly elevated the phosphorylation levels of LKB1, AMPK, and ACC in comparison with the H/R+ siRNA-NC group. Subsequently, results obtained from Figure 2d and 2e indicated that NR4A1 expression in Ov-NR4A1 group was dramatically enhanced when compared to that in the Ov-NC group. And overexpression of NR4A1 further suppressed the expression of LKB1/AMPK signaling in BRL-3A cells under H/R condition, evidenced by the decreased levels of p-LKB1, p-AMPK, and p-ACC (figure 2f). Overall, NR4A1 could significantly suppressed the expression of LKB1/AMPK pathway in BRL-3A cells treated with H/R.

### NR4A1-knockdown attenuates oxidative stress and inflammation of BRL-3A cells exposed to H/R through LKB1/AMPK pathway

**Figure 3.**
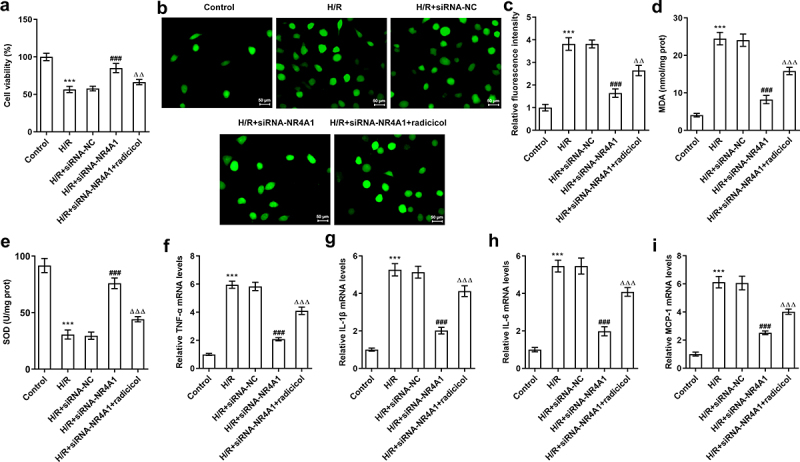
NR4A1-knockdown attenuates oxidative stress and inflammation of BRL-3A cells exposed to H/R through LKB1/AMPK pathway. (a) Cell viability was determined by CCK-8 assay. (b-c) Intracellular ROS level was examined by DCFH-DA staining. (d-e) Measurement of MDA and SOD by respective kits. (f-i) Expression levels of TNF-α, IL-1β, IL-6, and MCP-1 mRNA in BRL-3A cells treated with H/R was determined by RT-qPCR. ***P < 0.001 vs. control; ^###^P < 0.001 vs. H/R+ siRNA-NC; ^ΔΔ^P<0.01, ^ΔΔΔ^P<0.001 vs. H/R+ siRNA-NR4A1.

Next, radicicol, a LKB1 inhibitor, was used to treat BRL-3A cells to explore whether NR4A1-knockdown affected BRL-3A cell injury induced by H/R. As Figure 3a displayed, CCK-8 results showed that NR4A1 knockdown significantly increased the viability of BRL-3A cells treated with H/R, whereas radicicol could alleviate the promoting effect of NR4A1 on cell viability. Additionally, ROS staining results unraveled that radicicol alleviated the inhibitory effect of NR4A1 knockdown on ROS levels in H/R-induced BRL-3A cells (Figure 3b and 3c). Meanwhile, the elevated MDA content and reduced SOD activity in BRL-3A cells induced by H/R were remarkably improved after NR4A1 deficiency, whereas radicicol reversed the impacts of NR4A1 knockdown on MDA and SOD levels (Figure 3d and 3e). Moreover, RT-qPCR was used to determine the release of pro-inflammatory factors. As figure 3f-i suggested, radicicol decreased the inhibitory effect of NR4A1 knockdown on the expressions of TNF-α, IL-1β, IL-6, and MCP-1 in BRL-3A cells administrated with H/R. Altogether, NR4A1-knockdown alleviated the oxidative stress and inflammation of BRL-3A cells exposed to H/R through LKB1/AMPK pathway.

### NR4A1-knockdown ameliorates BRL-3A cell apoptosis induced by H/R through LKB1/AMPK pathway

**Figure 4.**
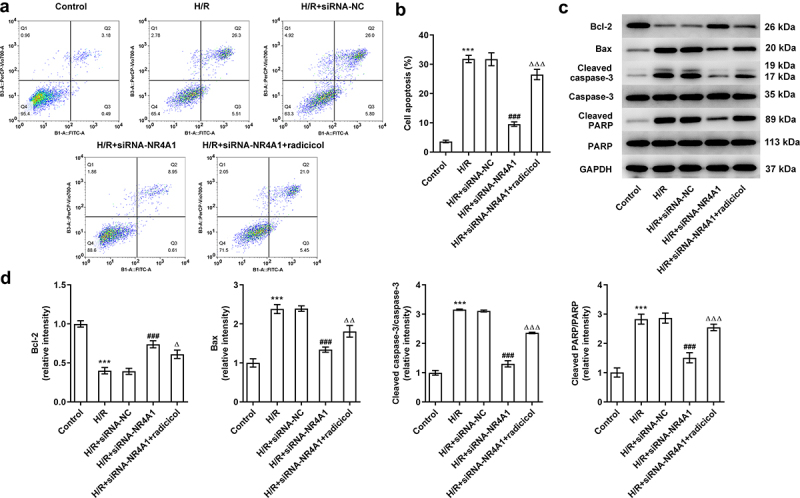
NR4A1-knockdown ameliorates BRL-3A cell apoptosis induced by H/R through LKB1/AMPK pathway. (a) Representative dot plots from flow cytometry analysis. (b) Cell apoptosis was determined by flow cytometry analysis. (c) Expression of apoptosis-related proteins (Bcl-2, Bax, caspase3, cleaved caspase3, PARP, and cleaved RAPR) were detected by western blotting. ***P < 0.001 vs. control; ^###^P < 0.001 vs. H/R+ siRNA-NC; ^Δ^P<0.05, ^ΔΔ^P<0.01, ^ΔΔΔ^P<0.001 vs. H/R+ siRNA-NR4A1.

Apoptosis acts as a critical player in IR-induced liver injury [15]. Therefore, flow cytometry and western blotting were conducted to determine the effect of NR4A1 on cell apoptosis induced by H/R through regulating LKB1/AMPK. As Figure 4a and 4b showed, cell apoptosis was hugely promoted in the H/R group in comparison with the control group, which was attenuated after NR4A1 deletion. Importantly, compared with the H/R+ siRNA-NR4A1 group, radicicol alleviated the inhibitory effect of NR4A1 knockdown on apoptosis. Moreover, results obtained from western blotting revealed that in contrast with the H/R+ siRNA-NC group, NR4A1 knockdown remarkably decreased the expression of anti-apoptotic protein Bcl-2 while increased the expression of pro-apoptotic proteins including Bax, cleaved caspase3, and cleaved RAPR. These changes could be reversed by radicicol (Figure 4c and 4d). These results implied that NR4A1-knockdown ameliorated BRL-3A cell apoptosis induced by H/R through LKB1/AMPK pathway.

### NR4A1-knockdown promotes autophagy of BRL-3A cells induced by H/R through LKB1/AMPK pathway

**Figure 5.**
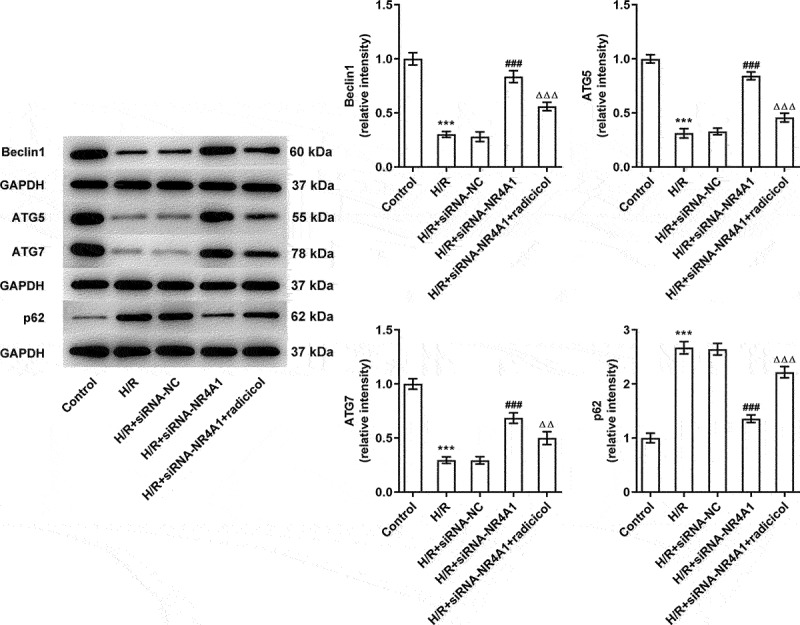
NR4A1-knockdown promotes autophagy of BRL-3A cells treated with H/R through LKB1/AMPK pathway. Western blot was used to evaluate the expression of autophagy-related proteins. ***P < 0.001 vs. control; ^###^P < 0.001 vs. H/R+ siRNA-NC; ^ΔΔ^P<0.01, ^ΔΔΔ^P<0.001 vs. H/R+ siRNA-NR4A1.

It has been reported that autophagy is caused by the upregulation of inflammatory mediators ROS or TNF-α and also plays an important role in the prevention of HIRI [16]. We also examined the changes of autophagy pathway-related proteins. As Figure 5 displayed, H/R exposure led to notably downregulated autophagy-related proteins Beclin1, ATG5, and ATG7 expression as well as upregulated p62 expression when compared to that in cells without any treatment. On the contrary, compared with the H/R group, NR4A1 knockdown promoted the contents of Beclin1, ATG5, and ATG7 while suppressed the expression of p62, indicated that NR4A1 knockdown could promote autophagy in H/R-induced BRL-3A cells. Likewise, compared with the H/R+ siRNA-NR4A1 group, radicicol conspicuously diminished the levels of Beclin1, ATG5, and ATG7 expression but enhanced p62 expression. In summary, downregulation of NR4A1 could promote autophagy of BRL-3A cells induced by H/R by activating LKB1/AMPK pathway.

## Discussion

HIRI is the main cause of poor prognosis in liver surgery [17]. Current studies have shown that the pathological process of HIRI is closely related to the activation of innate immune system, apoptosis, the release of ROS and inflammation [4]. As the most important metabolic regulatory organ, liver serves as a vital player in anti-oxidation. When the IRI occurs, the body is in a state of stress which affects the antioxidant capacity of liver and reduces the antioxidant stress capacity of the body [18]. Meanwhile, a large number of inflammatory factors are released into the blood and act on the liver to cause a series of pathological injuries [19]. Apoptosis is also another important mechanism of liver injury caused by H/R [20,21]. In addition, autophagy is caused by the upregulation of inflammatory mediators ROS or TNF-α and also plays an important role in the prevention of HIRI [16]. Therefore, in the present study, we focused on the effects of NR4A1 on oxidative stress, inflammatory cytokines, apoptosis, and autophagy in ischemia-reperfusion hepatocytes. Our results showed that NR4A1 was upregulated in BRL-3A cells stimulated with H/R, and downregulation of NR4A1 could improve BRL-3A cell injury by activating LKB1/AMPK pathway.

Being a class of ligand-dependent transcription factors, NR4A1 affects cell cycle, inflammatory response, and apoptosis by binding to corresponding ligands to regulate the expression of gene networks [^22–24^]. Large studies showed that NR4A1 plays an important role in the progression of various tumors. For example, in lymphoma, overexpression of NR4A1 could induce apoptosis and then reduce the tumor growth [25]. In breast cancer, the abnormal expression of NR4A1 imparts suppressive on the proliferative and migrative abilities of cancer cells [26]. Recent studies have shown that NR4A1 is closely related to IRI in multiple tissues/organs. The expression of NR4A1 was upregulated both in mouse liver tissue treated with IRI and microglia after cerebral IRI indicates that NR4A1 contributes to the promotion of IRI [6,8]. In addition, NR4A1 aggravates cardiac microvascular IRI through suppressing Fundc1-mediated mitochondrial autophagy [27]. In the present study, NR4A1 was highly expressed in BRL-3A cells treated with H/R. In the HIRI model, NR4A1 knockdown significantly enhanced cell activity and autophagy pathway activation (autophagy-related proteins including the upregulation of Beclin1, ATG5, and ATG7 and the downregulation of p62), whereas suppressed inflammation, oxidative stress, as well as apoptosis.

To further explore the downstream regulation mechanism of NR4A1 in H/R-induced hepatocyte damage, we found that LKB1/AMPK pathway may serve as the downstream regulatory pathway of NR4A1. LKB-1 protein is widely distributed in the cells of human tissues and plays an important role. Studies have shown that LKB1 could phosphorylate AMP in a MPK-dependent manner, which is an important upstream kinase of AMPK [28,29]. In addition, ROS could indirectly activate LKB1/AMPK pathway through phosphorylating ATM and promote autophagy [30]. Interestingly, the inhibition of NR4A1 could enhance the activation of LKB1/AMPK pathway [31]. Meanwhile, activated AMPK may have a protective effect on HIRI by preventing energy decrease, inhibiting inflammation, inhibiting hepatocyte apoptosis, and reducing oxidative stress [32,33]. And autophagy induction can be determined by different proteins, such as LKB1 and AMPK [34]. In the present study, following the BRL-3A cells treated with H/R, the LKB1/AMPK pathway was suppressed. Notably, NR4A1 knockdown could improve the viability and autophagy, and inhibited the oxidative stress, inflammation and apoptosis by activating LKB1/AMPK pathway, indicating that the ameliorative effect of NR4A1 on HIRI was achieved through regulation of LKB1/AMPK pathway.

In conclusion, our study demonstrated that NR4A1 was highly expressed in HIRI cell model. NR4A1 knockdown could enhance BRL-3A cell viability and autophagy, whereas inhibiting inflammation, oxidative stress and apoptosis by activating LKB1/AMPK pathway, providing evidence that the protection role and specific mechanism of NR4A1 knockdown in HIRI. Our finding may offer a promising target and open novel avenues for future therapies of HIRI.

## Supplementary Material

Supplemental Material

## Data Availability

All data included in this study are available upon request through contact with the corresponding author.
