# Supplementary figures and images for: Nuclear receptor 4A1 (NR4A1) silencing protects hepatocyte against hypoxia-reperfusion injury in vitro by activating liver kinase B1 (LKB1)/AMP-activated protein kinase (AMPK) signaling
Source: Bioengineered. 2022 Mar 21;13(4):8349–59. doi: 10.1080/21655979.2022.2053804 (PMC9161842; doi:10.1080/21655979.2022.2053804)

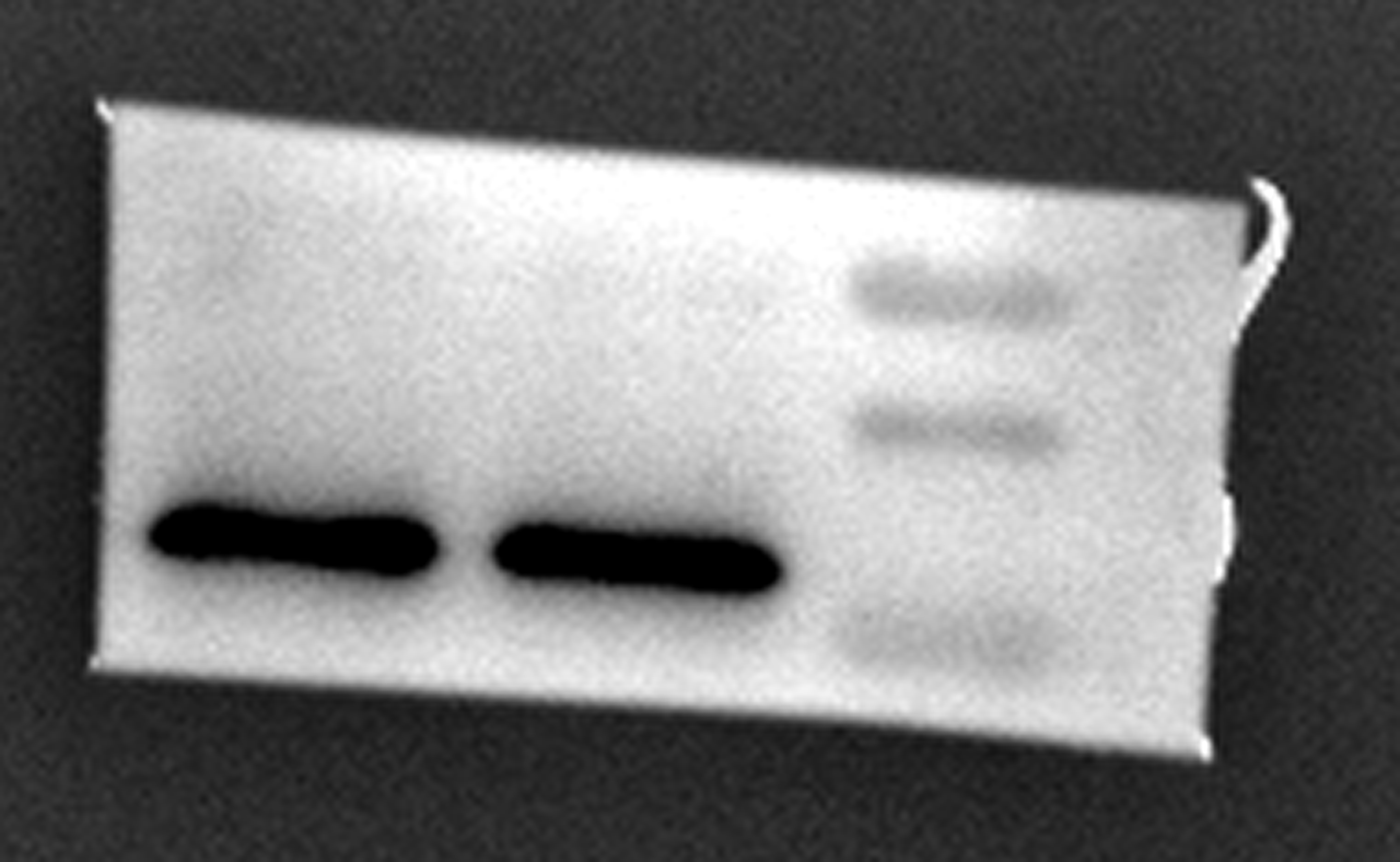

Supplement: Supplemental Material [file KBIE_A_2053804_SM3009.zip › Fig1A_GAPDH.tif]

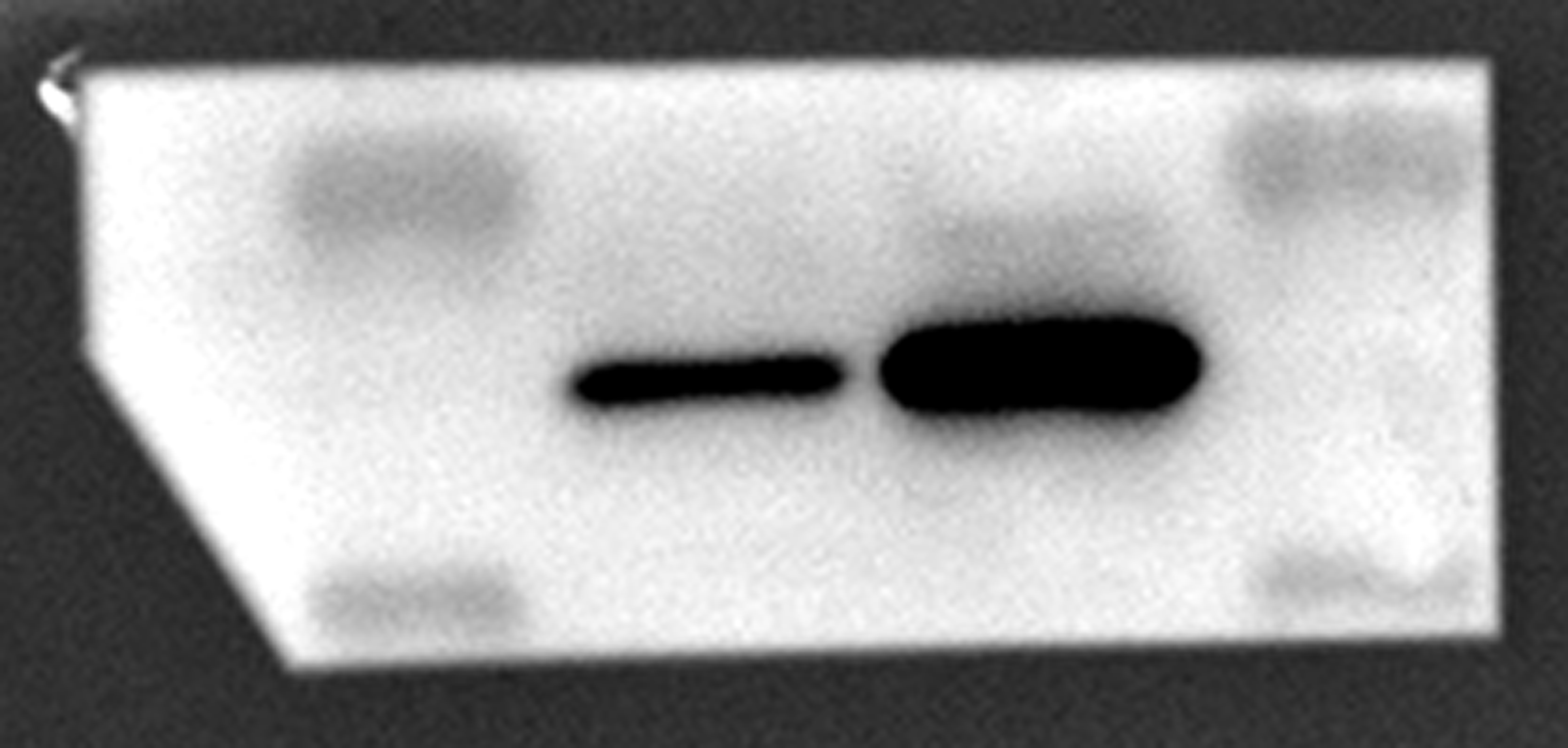

Supplement: Supplemental Material [file KBIE_A_2053804_SM3009.zip › Fig1A_NR4A1.tif]

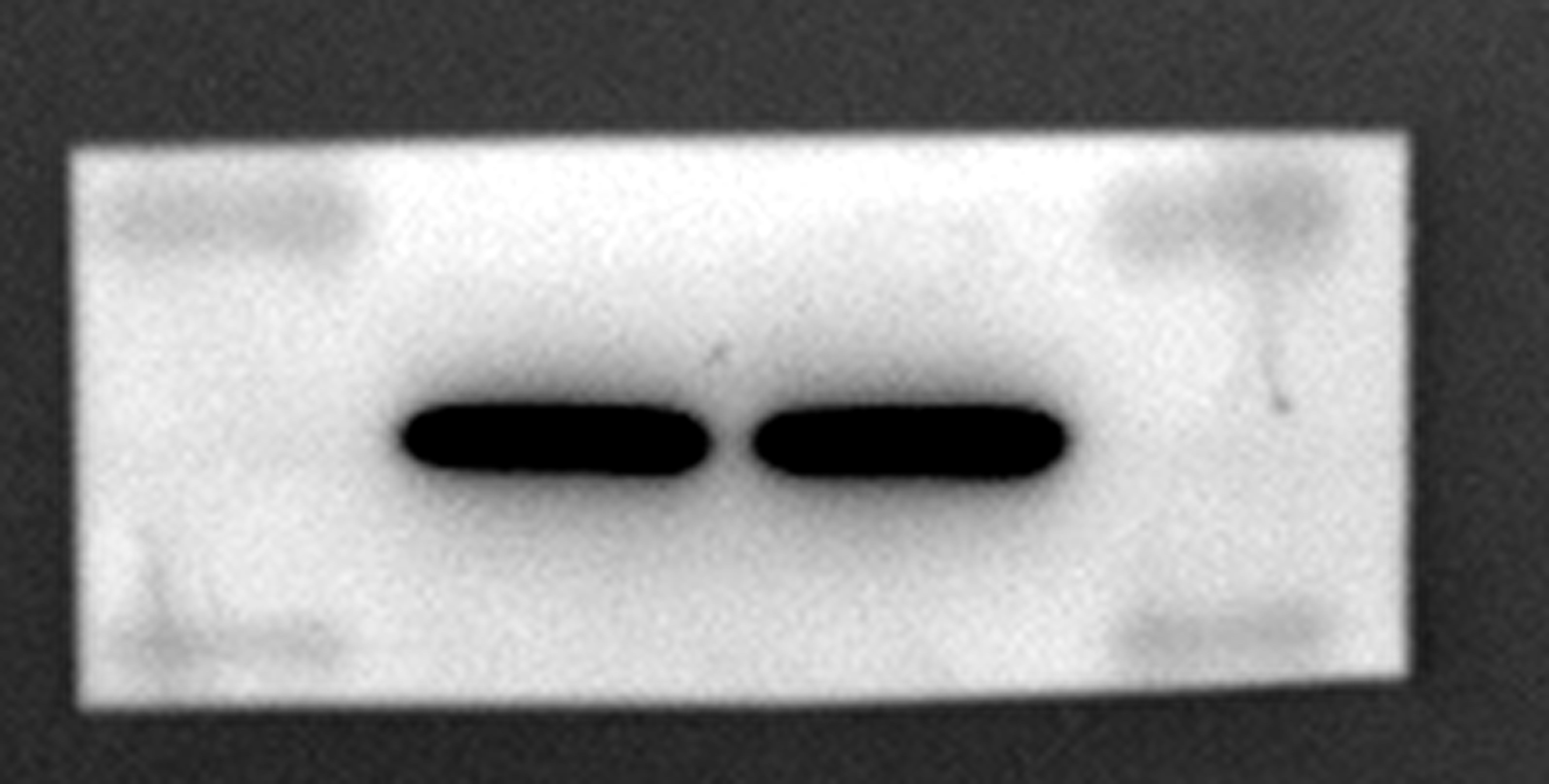

Supplement: Supplemental Material [file KBIE_A_2053804_SM3009.zip › Fig1C_ACC.tif]

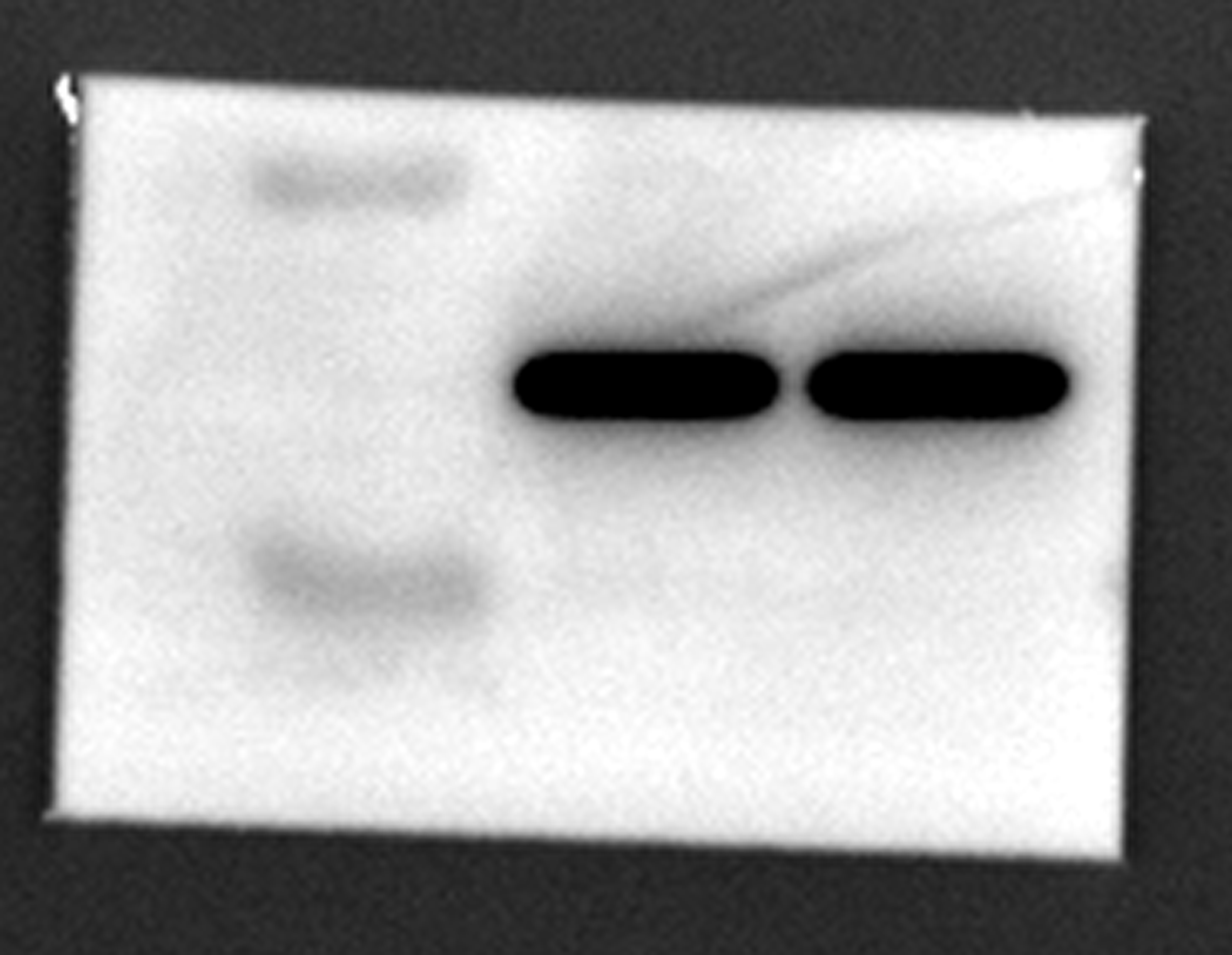

Supplement: Supplemental Material [file KBIE_A_2053804_SM3009.zip › Fig1C_AMPK.tif]

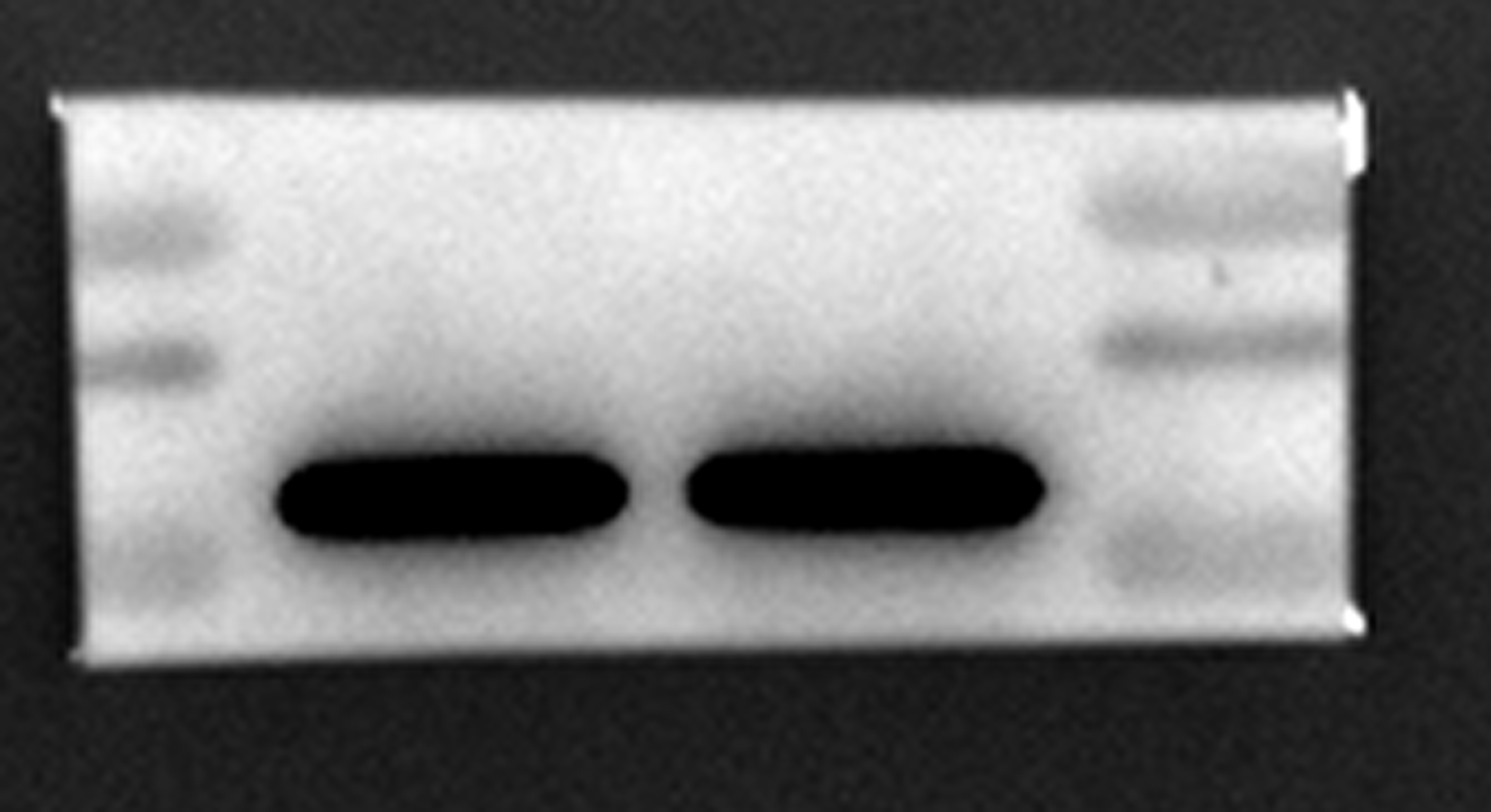

Supplement: Supplemental Material [file KBIE_A_2053804_SM3009.zip › Fig1C_GAPDH.tif]

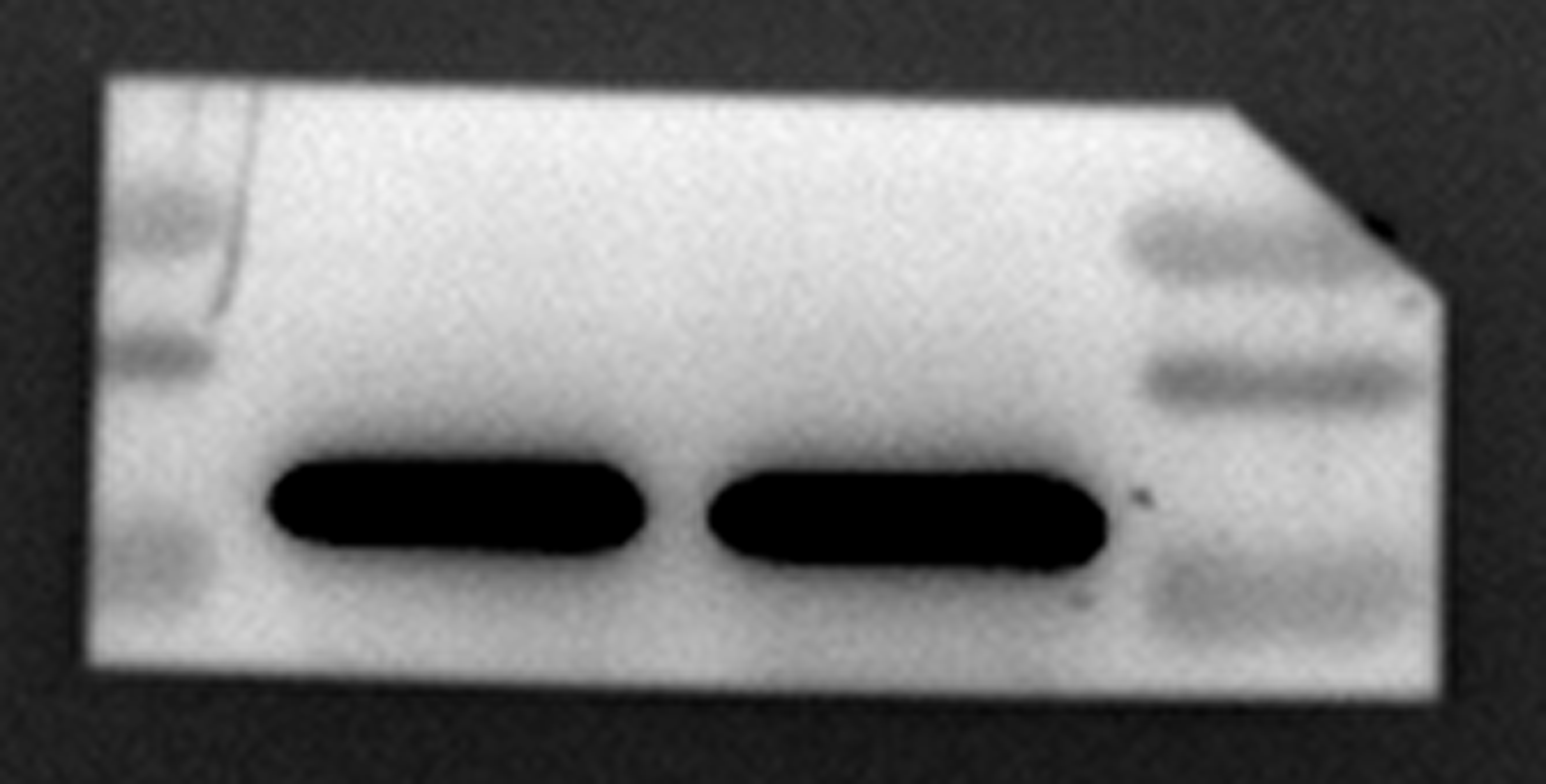

Supplement: Supplemental Material [file KBIE_A_2053804_SM3009.zip › Fig1C_GAPDH_1.tif]

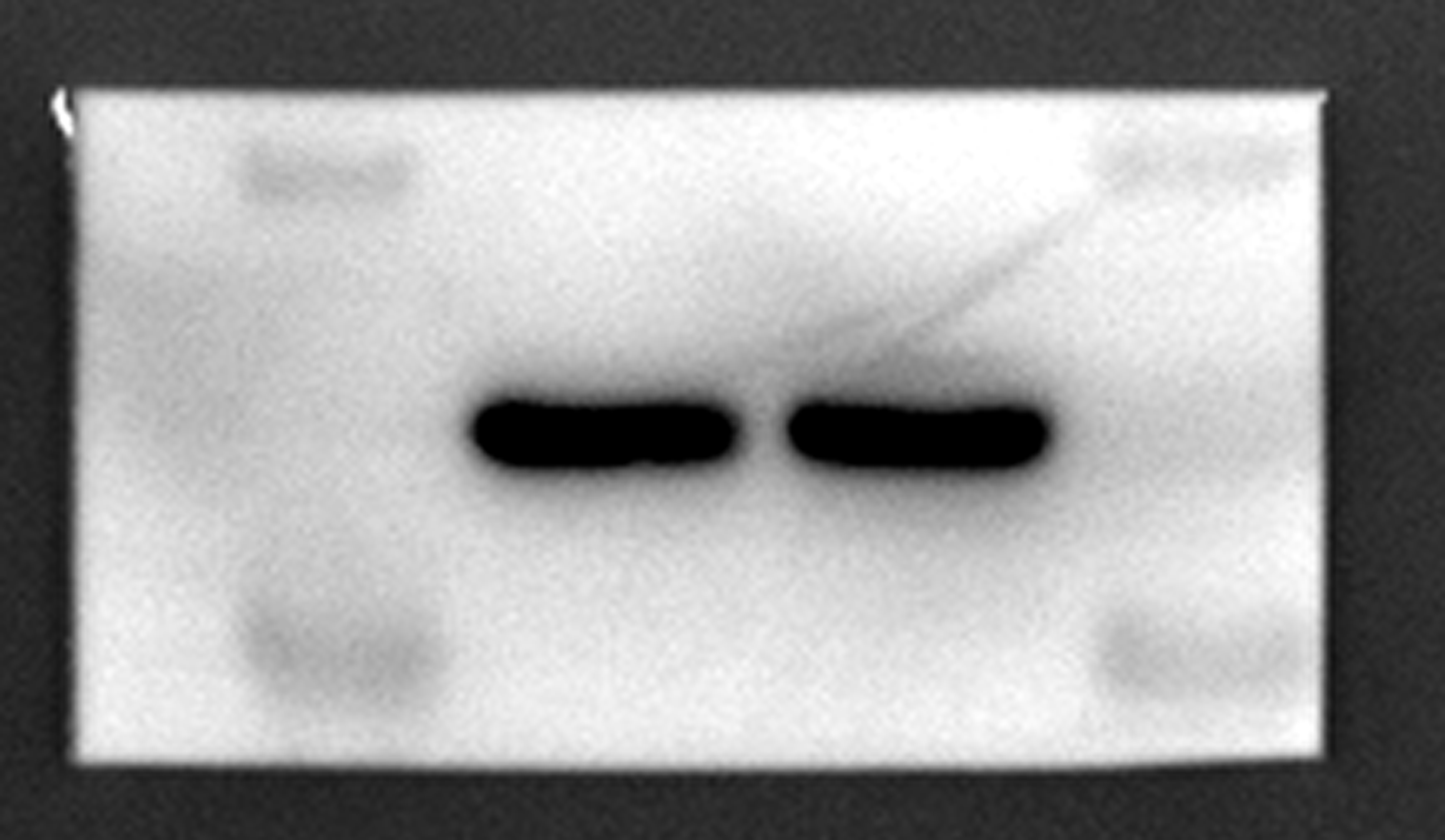

Supplement: Supplemental Material [file KBIE_A_2053804_SM3009.zip › Fig1C_LKB1.tif]

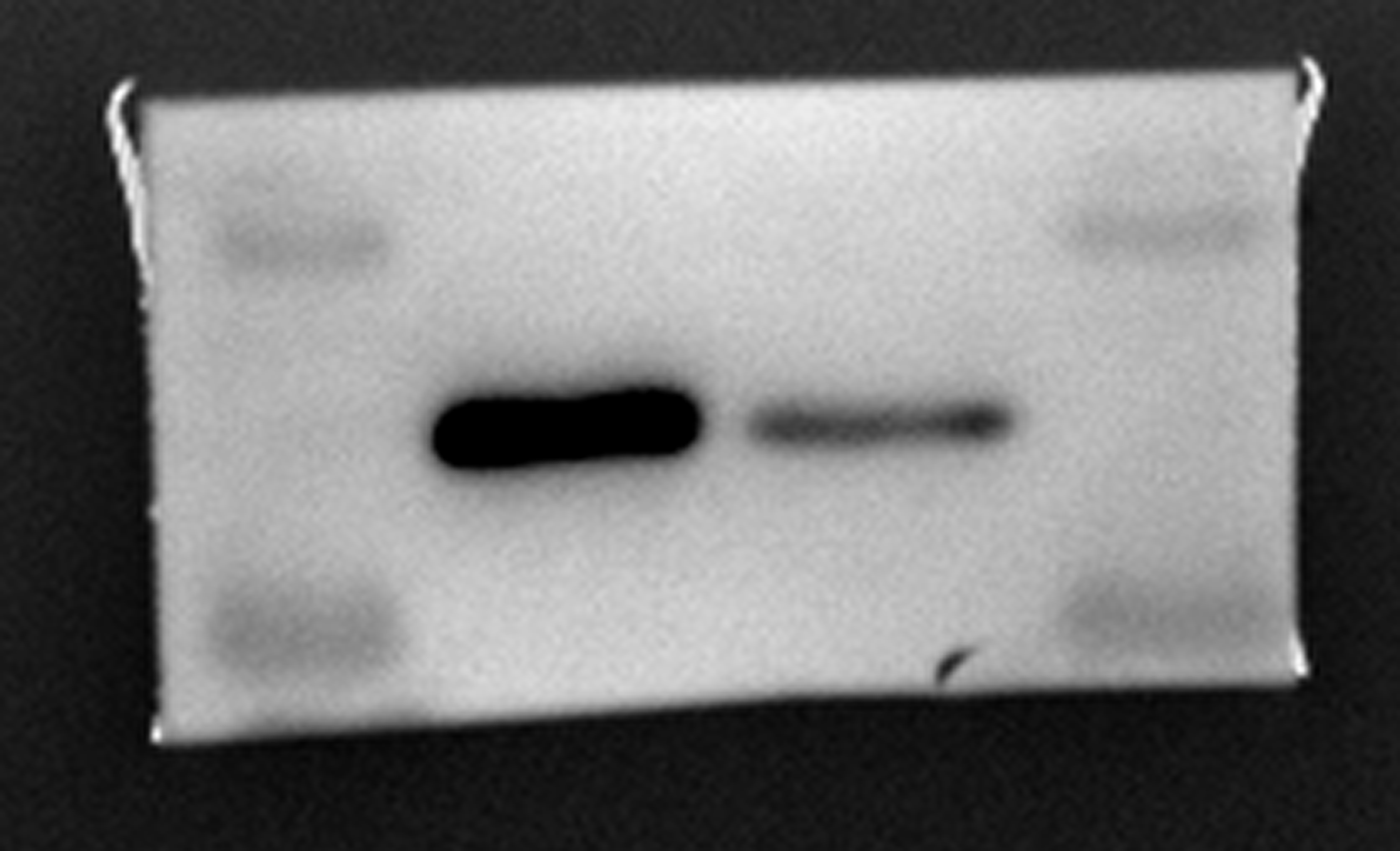

Supplement: Supplemental Material [file KBIE_A_2053804_SM3009.zip › Fig1C_p_ACC.tif]

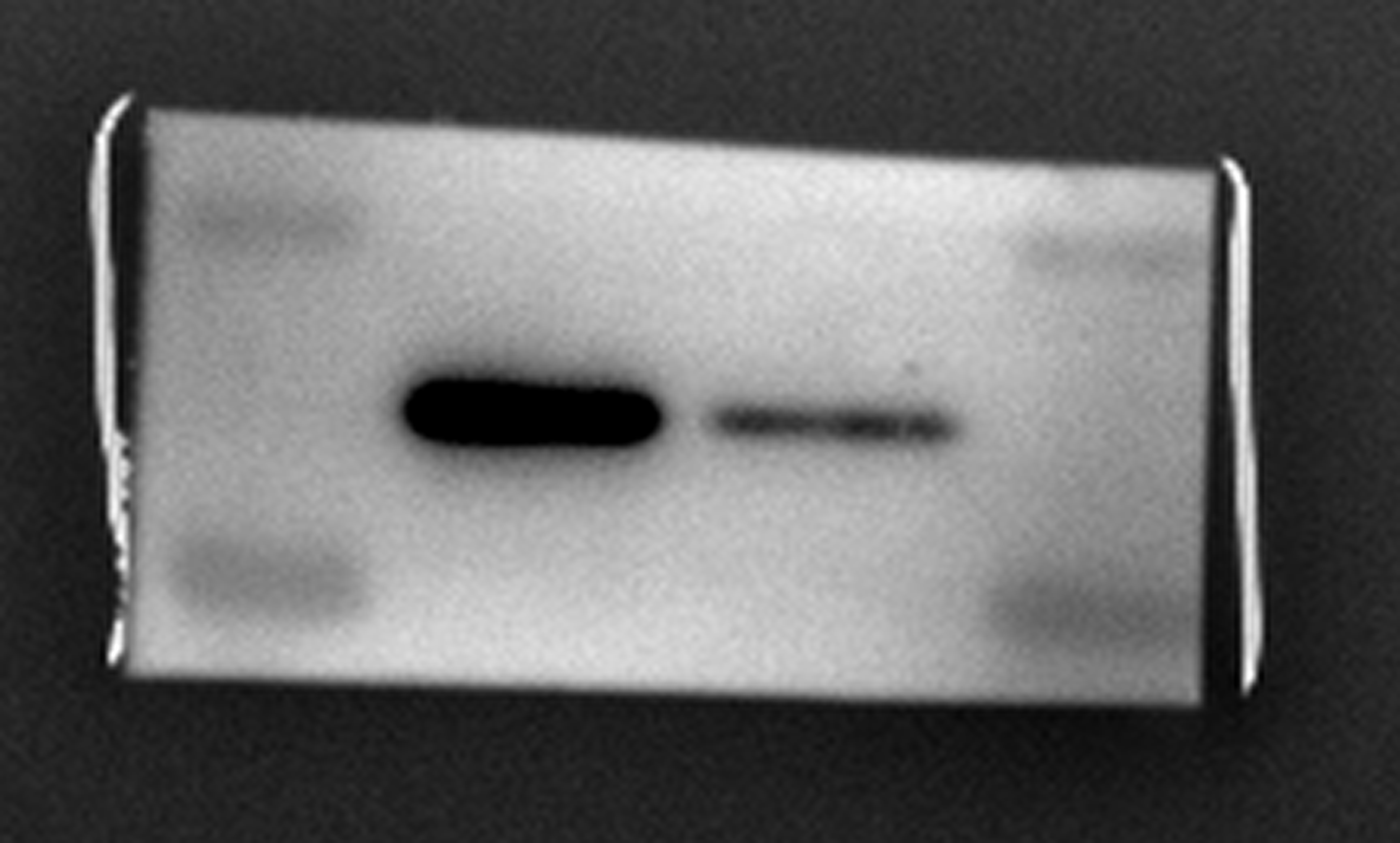

Supplement: Supplemental Material [file KBIE_A_2053804_SM3009.zip › Fig1C_p_AMPK.tif]

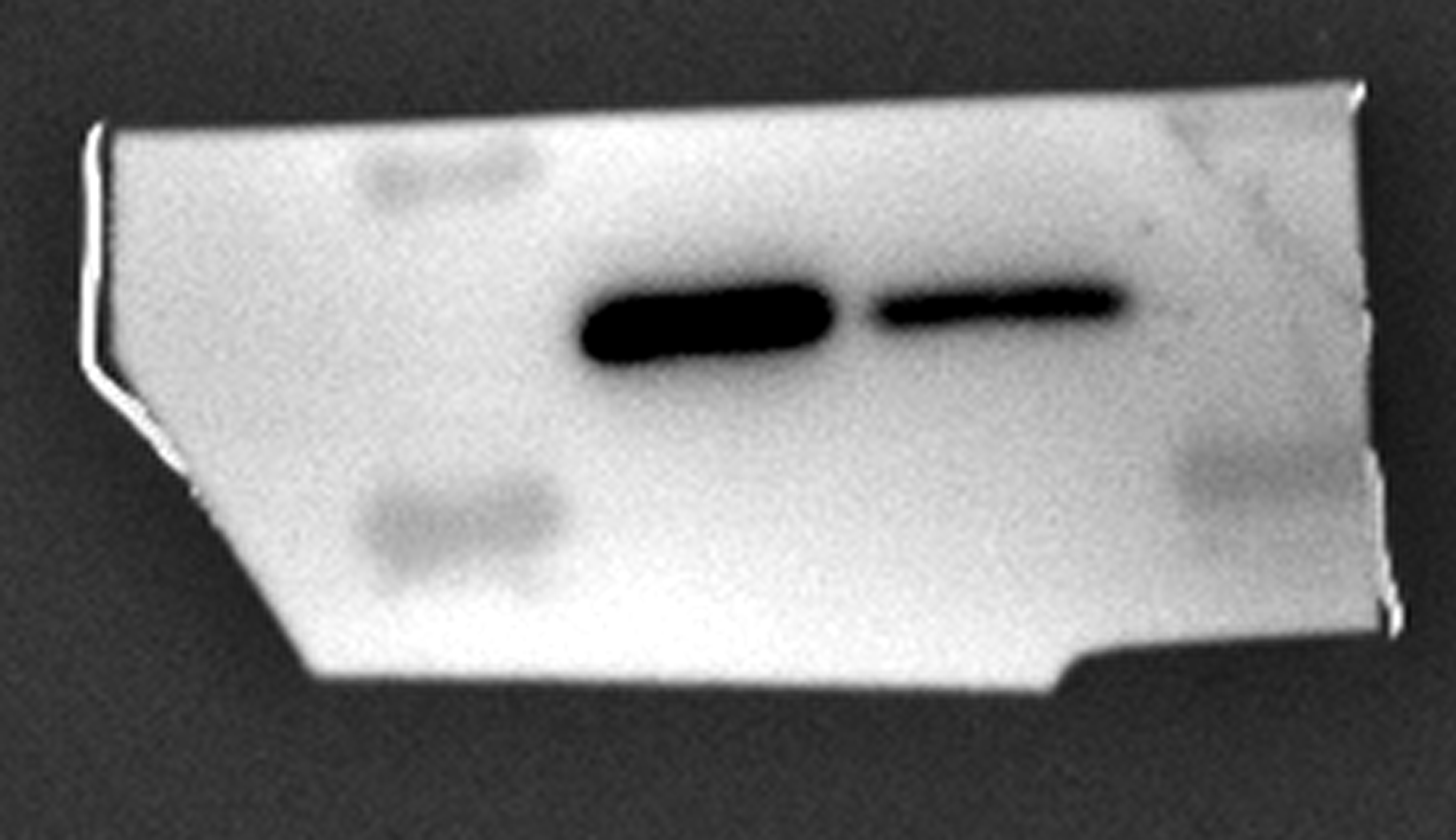

Supplement: Supplemental Material [file KBIE_A_2053804_SM3009.zip › Fig1C_p_LKB1.tif]

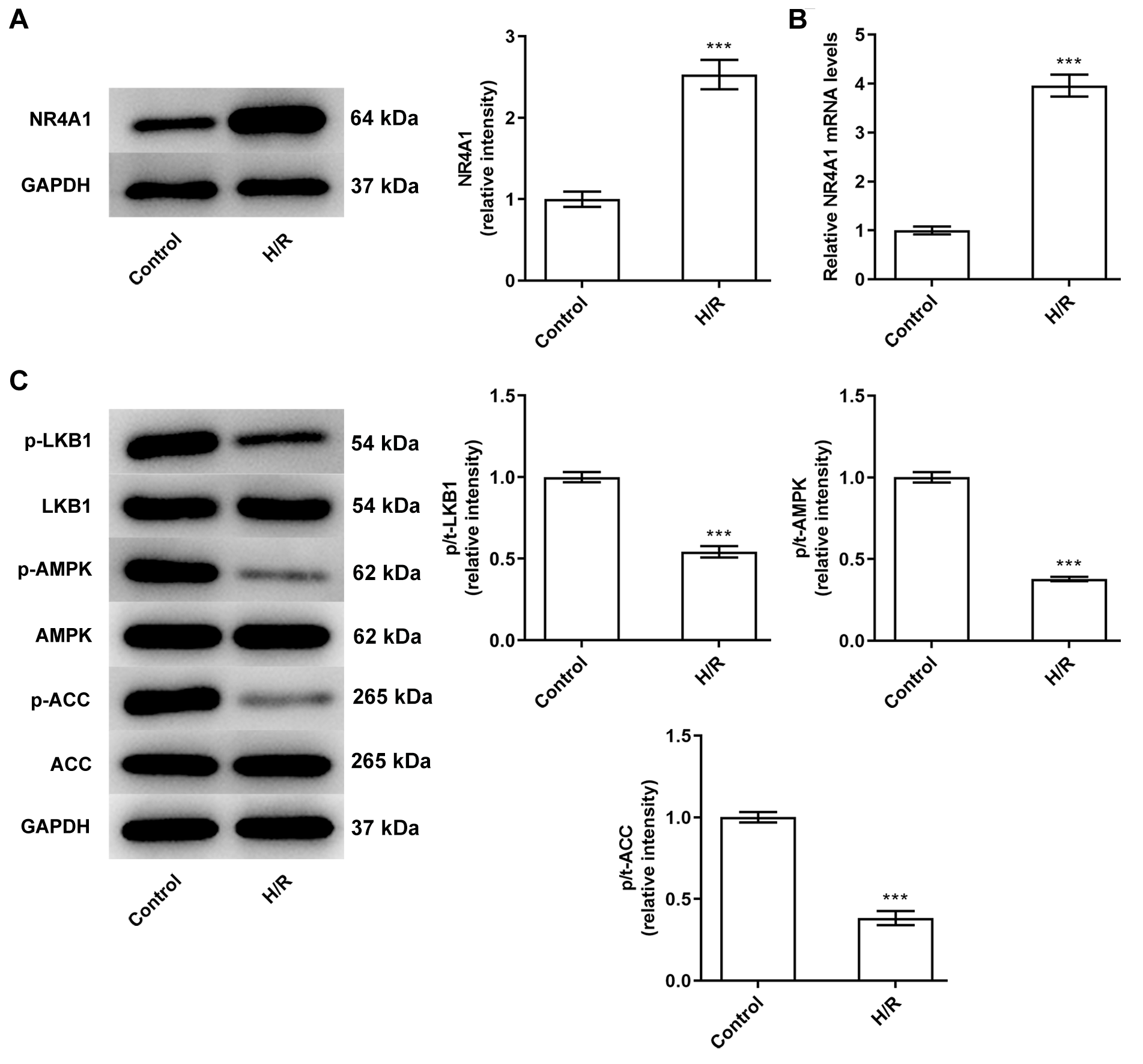

Supplement: Supplemental Material [file KBIE_A_2053804_SM3009.zip › fig1_revised.tif]

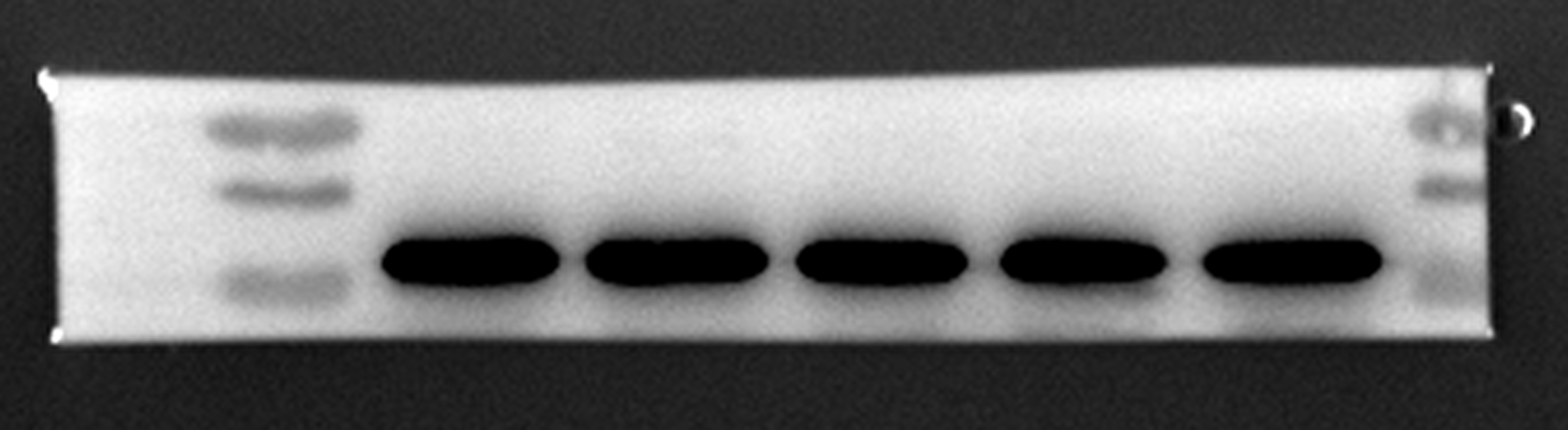

Supplement: Supplemental Material [file KBIE_A_2053804_SM3009.zip › Fig2A_GAPDH.tif]

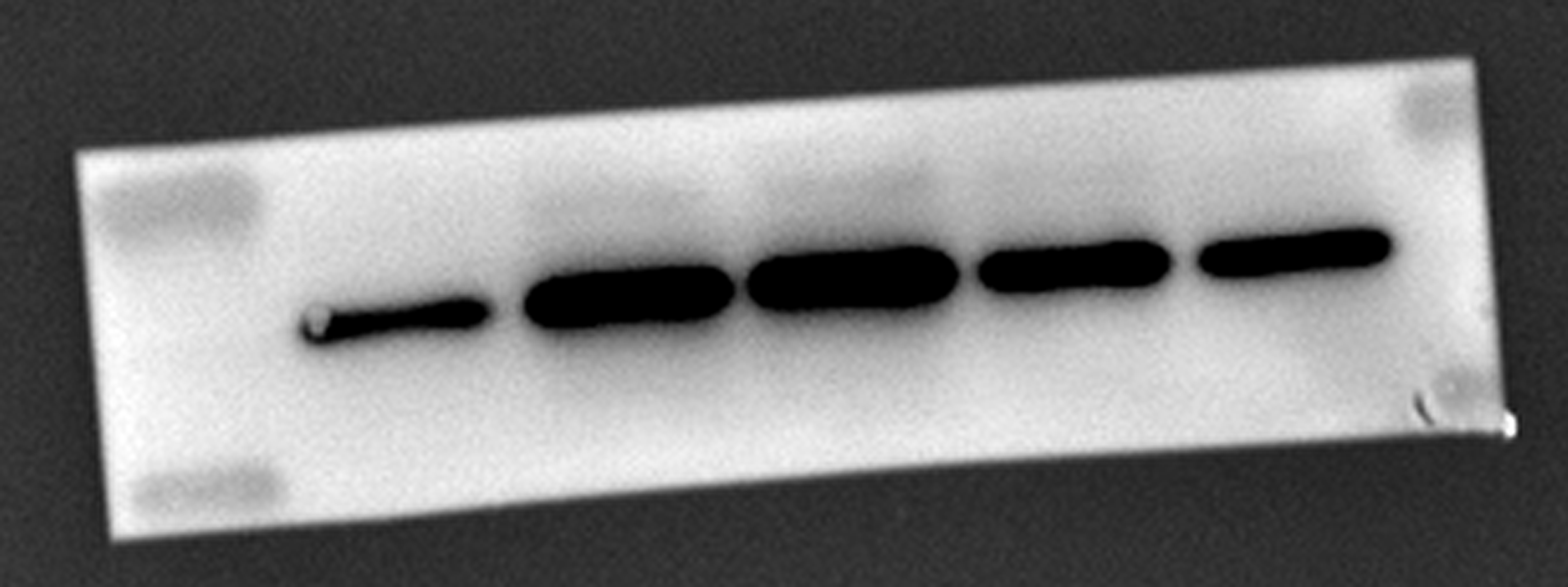

Supplement: Supplemental Material [file KBIE_A_2053804_SM3009.zip › Fig2A_NR4A1.tif]

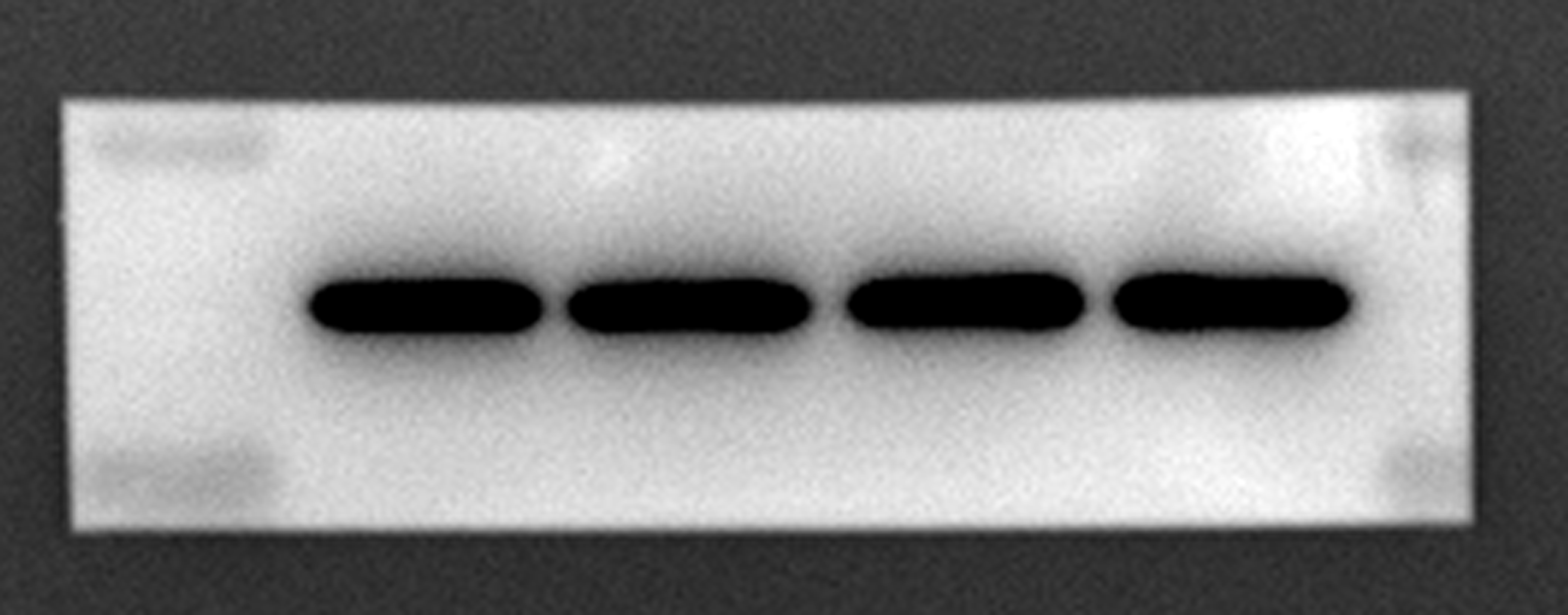

Supplement: Supplemental Material [file KBIE_A_2053804_SM3009.zip › Fig2C_ACC.tif]

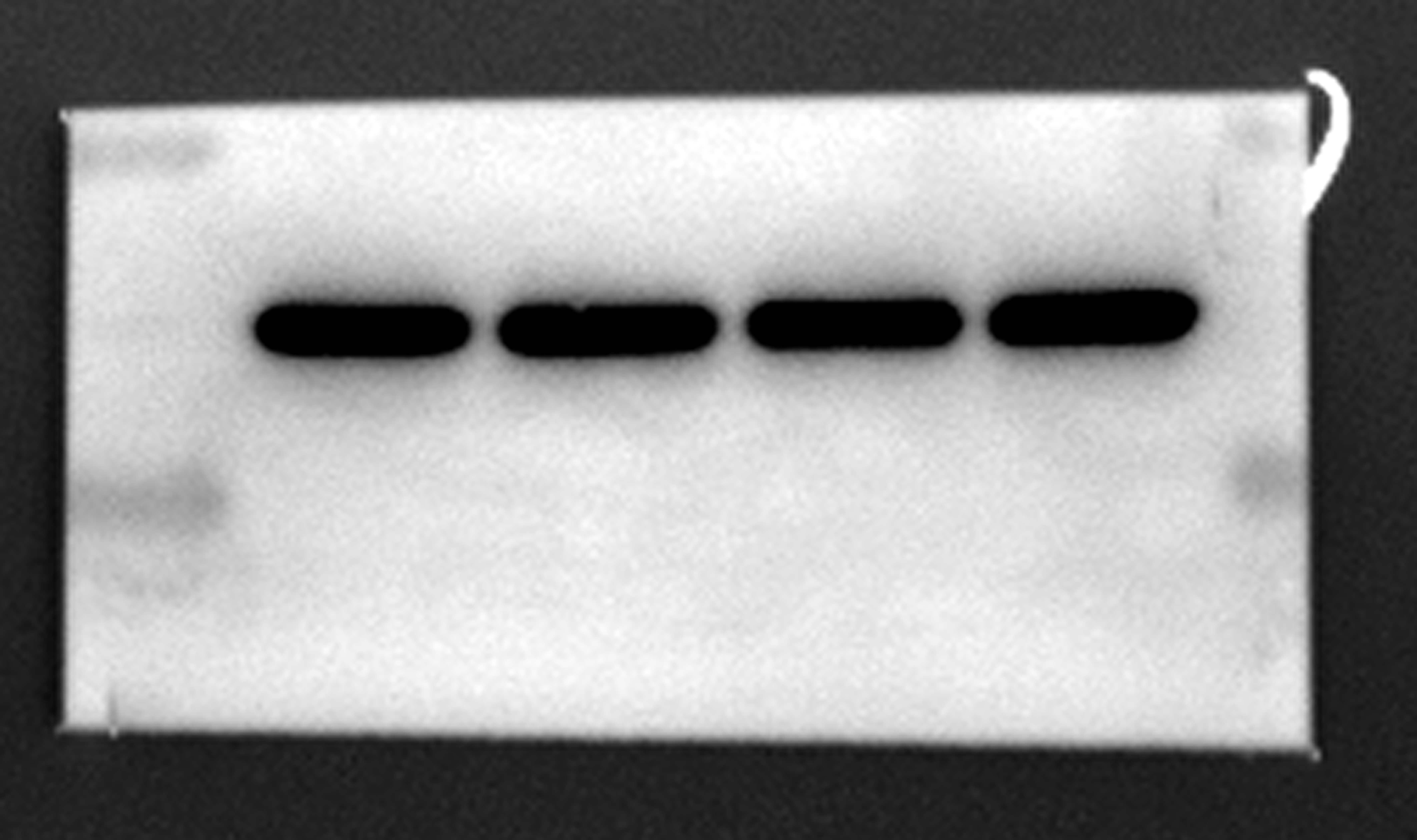

Supplement: Supplemental Material [file KBIE_A_2053804_SM3009.zip › Fig2C_AMPK.tif]

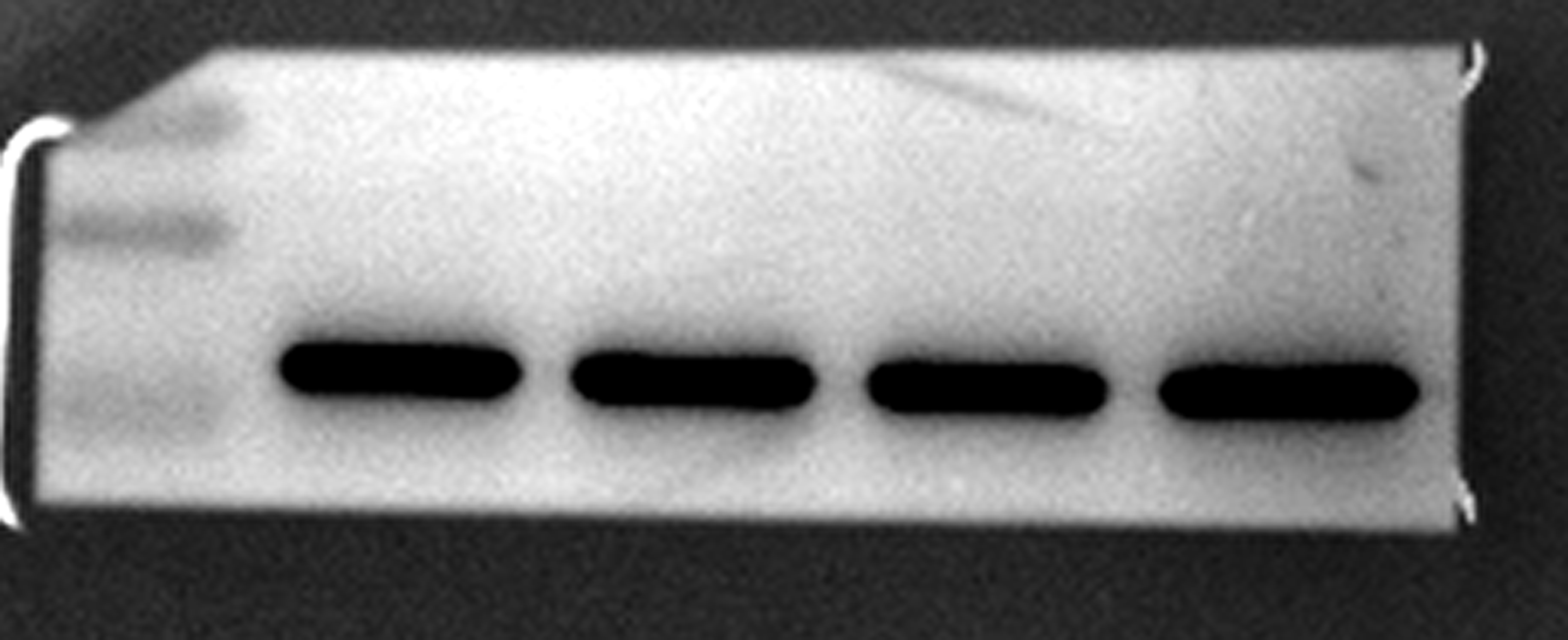

Supplement: Supplemental Material [file KBIE_A_2053804_SM3009.zip › Fig2C_GAPDH.tif]

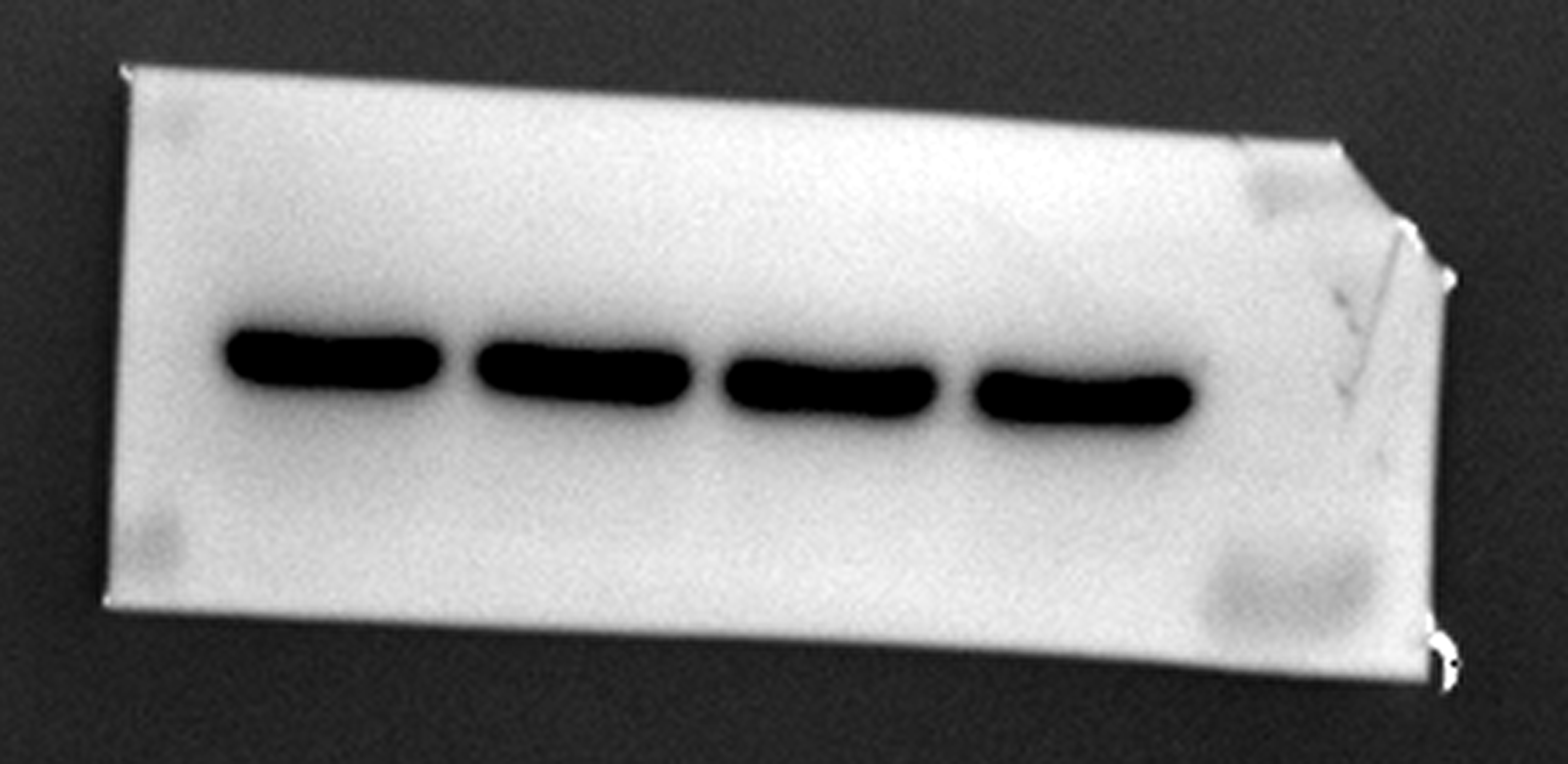

Supplement: Supplemental Material [file KBIE_A_2053804_SM3009.zip › Fig2C_LKB1.tif]

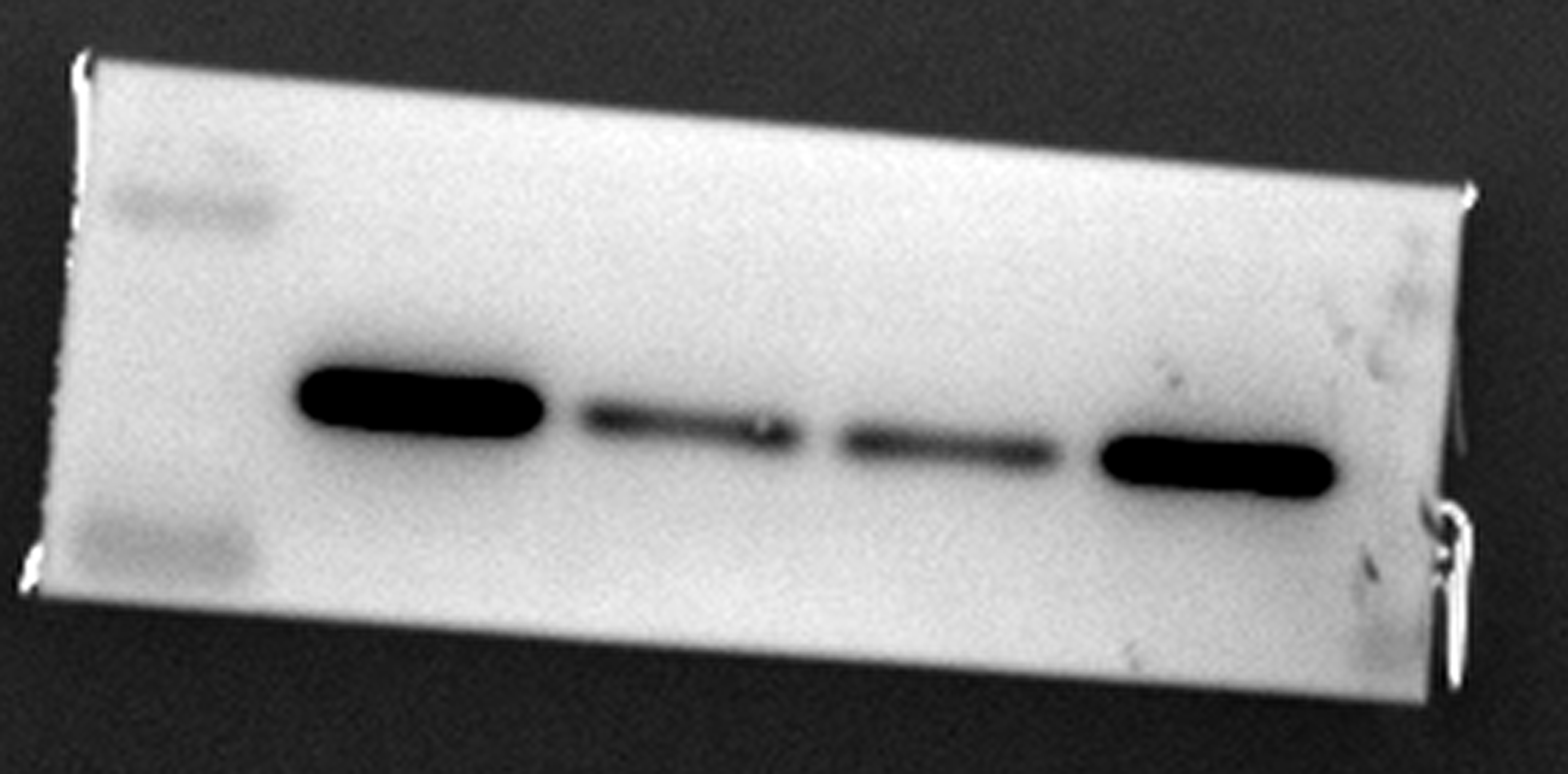

Supplement: Supplemental Material [file KBIE_A_2053804_SM3009.zip › Fig2C_p_ACC.tif]

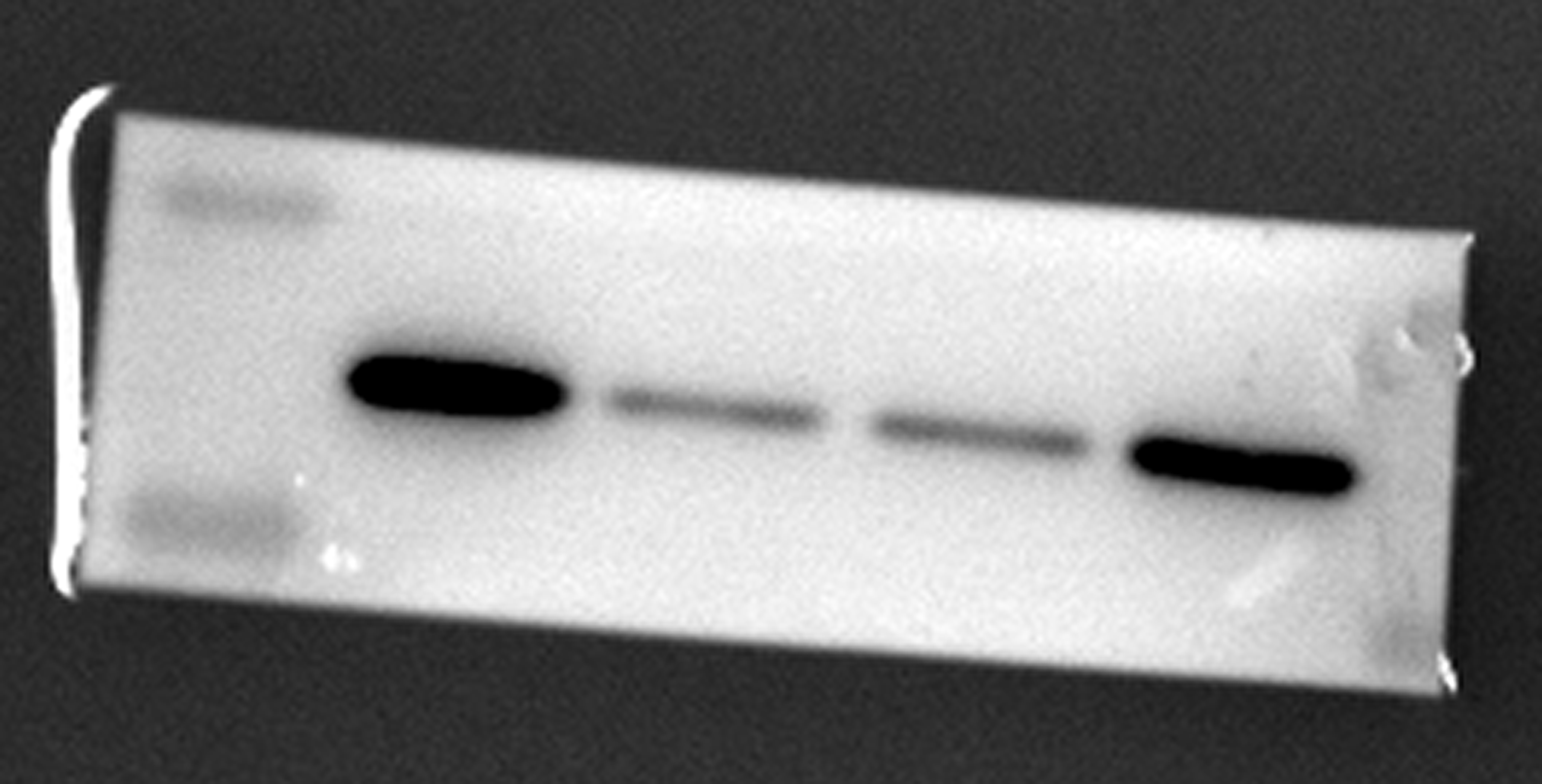

Supplement: Supplemental Material [file KBIE_A_2053804_SM3009.zip › Fig2C_p_AMPK.tif]

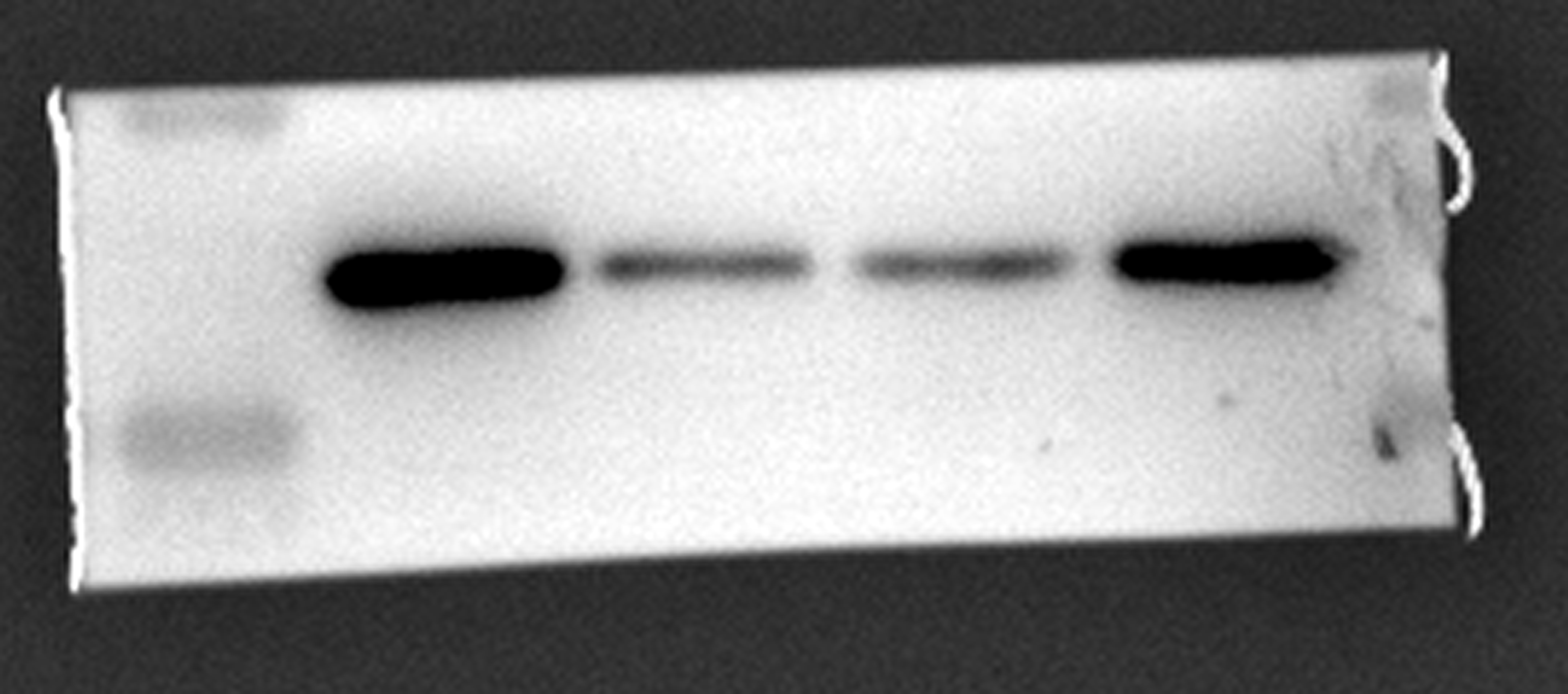

Supplement: Supplemental Material [file KBIE_A_2053804_SM3009.zip › Fig2C_p_LKB1.tif]

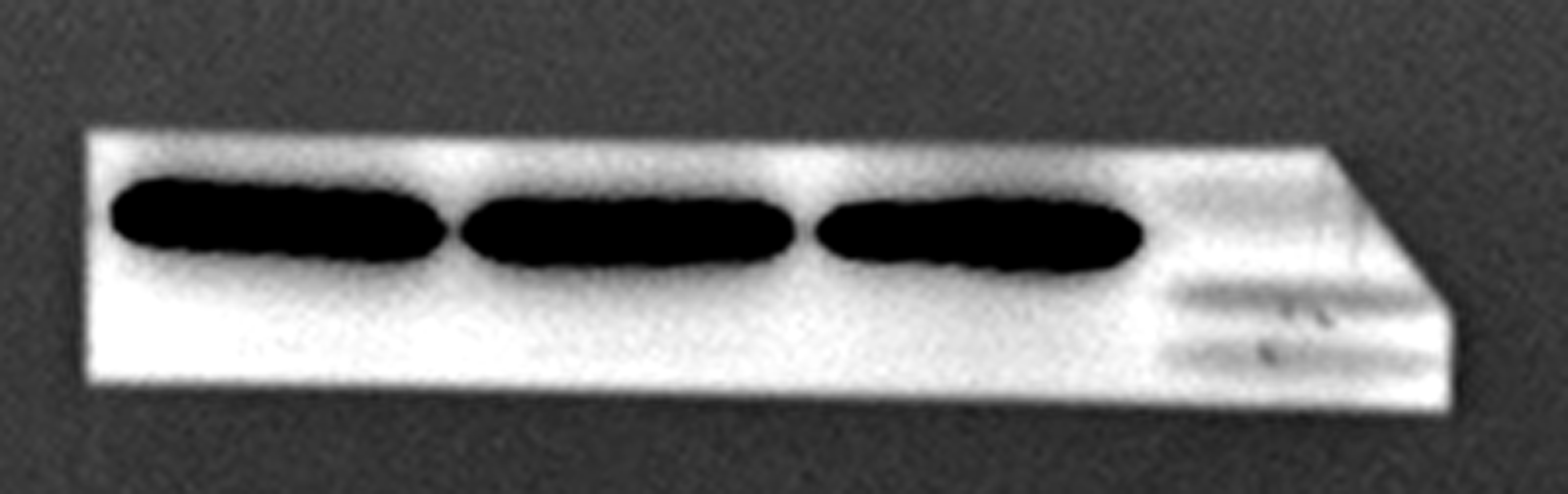

Supplement: Supplemental Material [file KBIE_A_2053804_SM3009.zip › Fig2D_GAPDH.tif]

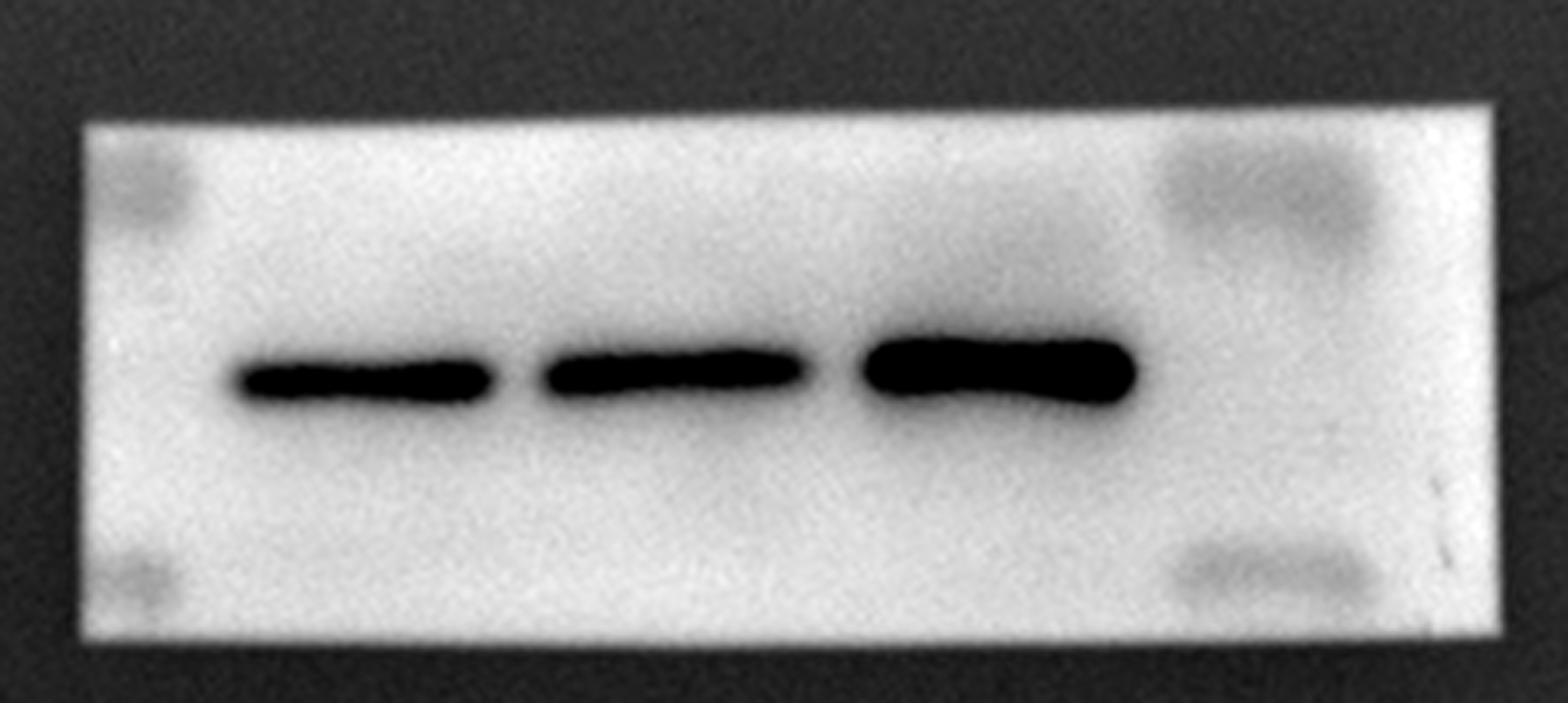

Supplement: Supplemental Material [file KBIE_A_2053804_SM3009.zip › Fig2D_NR4A1.tif]

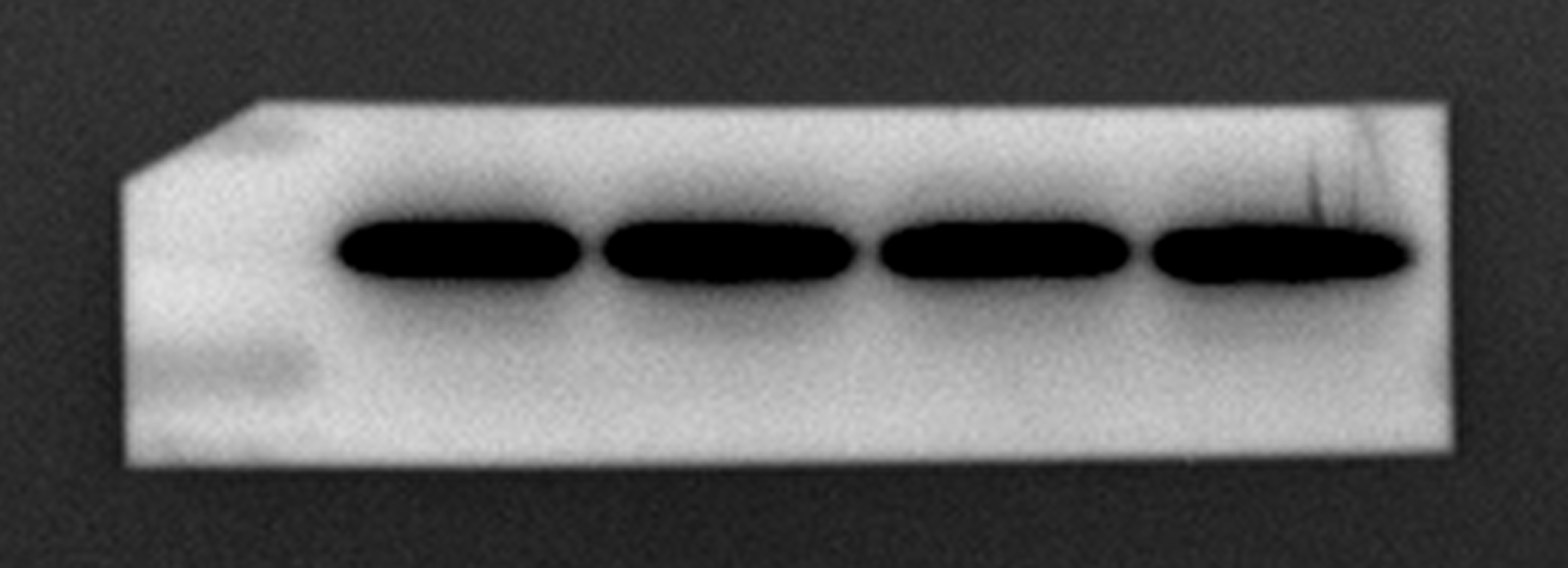

Supplement: Supplemental Material [file KBIE_A_2053804_SM3009.zip › Fig2F_ACC.tif]

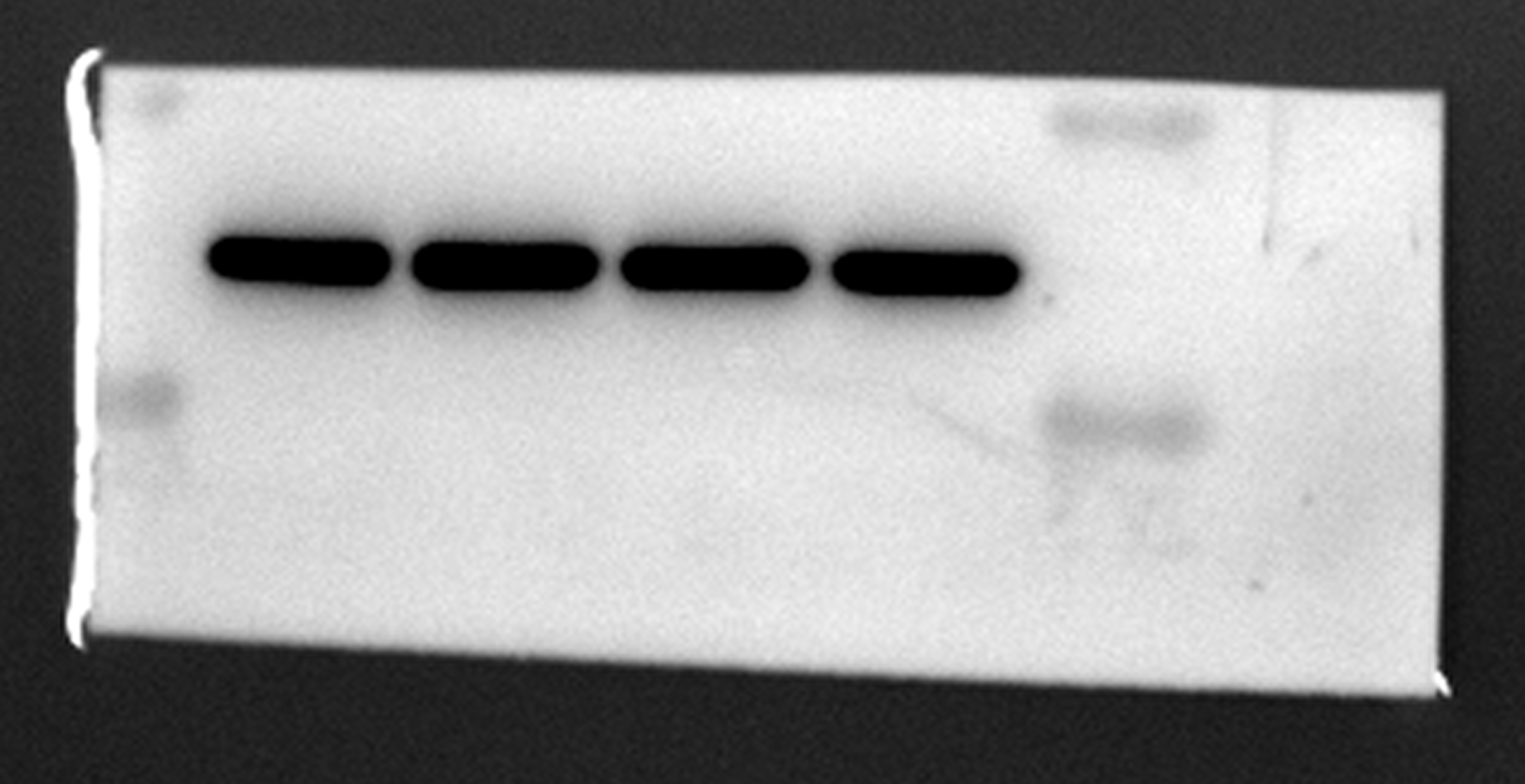

Supplement: Supplemental Material [file KBIE_A_2053804_SM3009.zip › Fig2F_AMPK.tif]

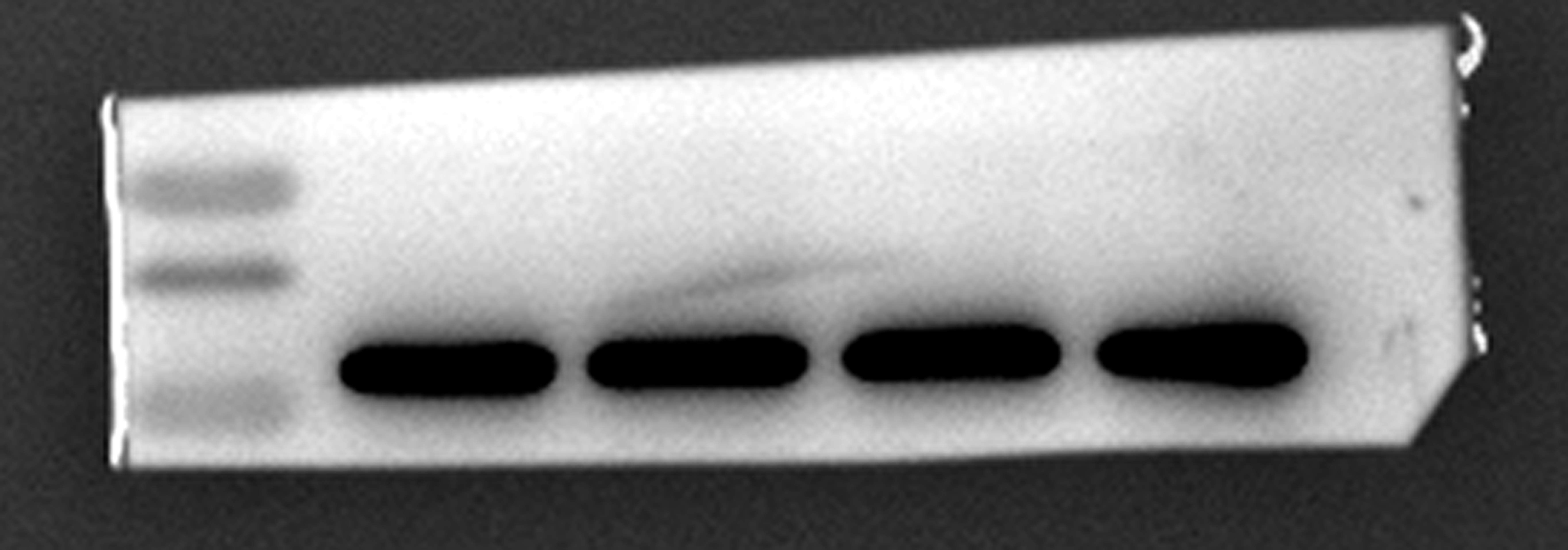

Supplement: Supplemental Material [file KBIE_A_2053804_SM3009.zip › Fig2F_GAPDH.tif]

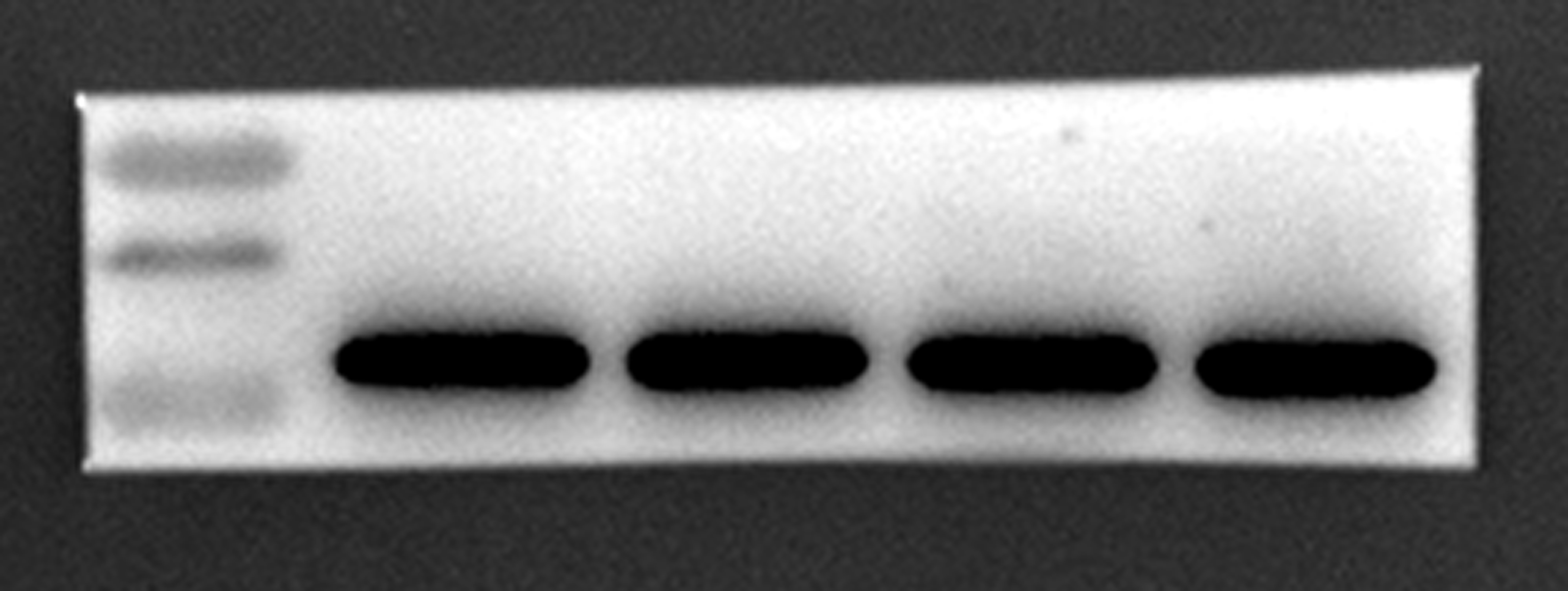

Supplement: Supplemental Material [file KBIE_A_2053804_SM3009.zip › Fig2F_GAPDH_1.tif]

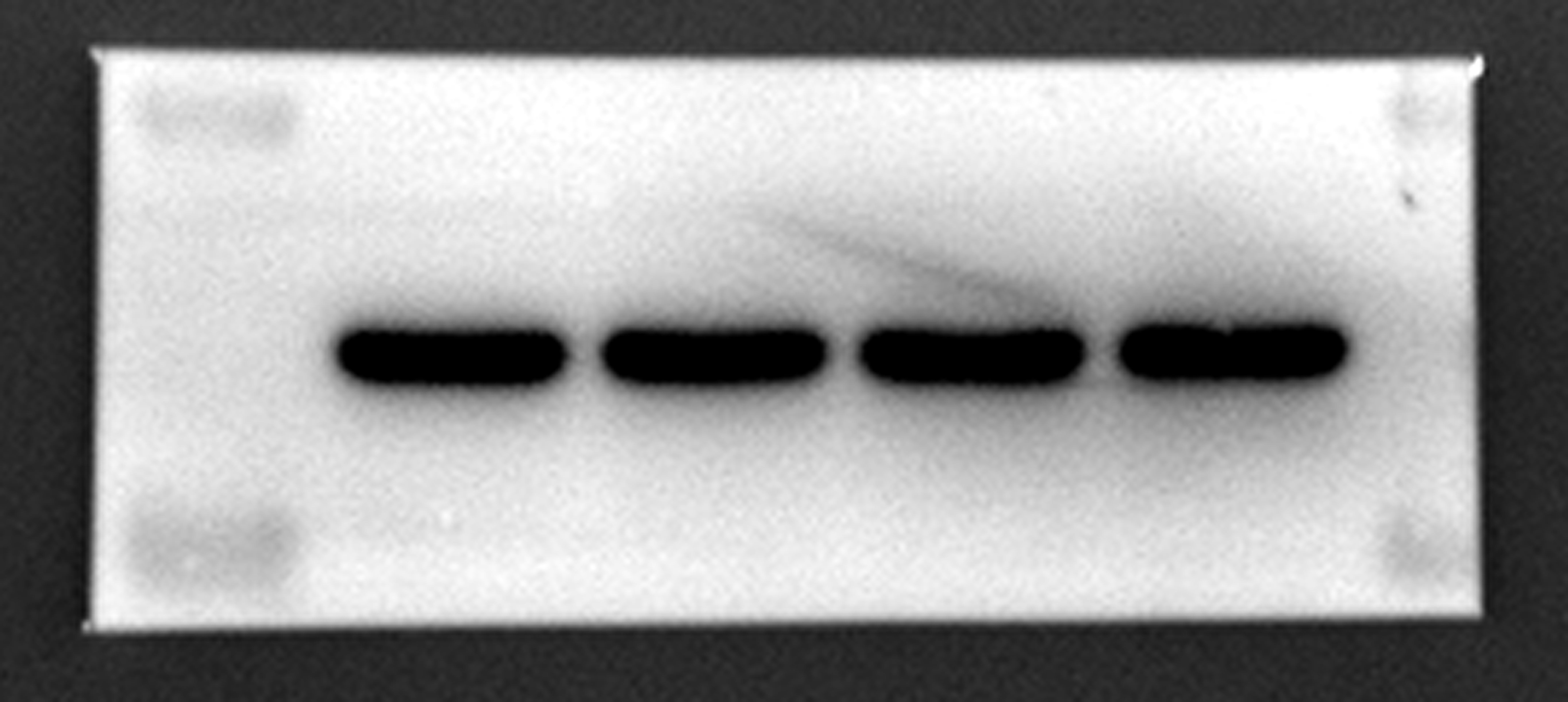

Supplement: Supplemental Material [file KBIE_A_2053804_SM3009.zip › Fig2F_LKB1.tif]

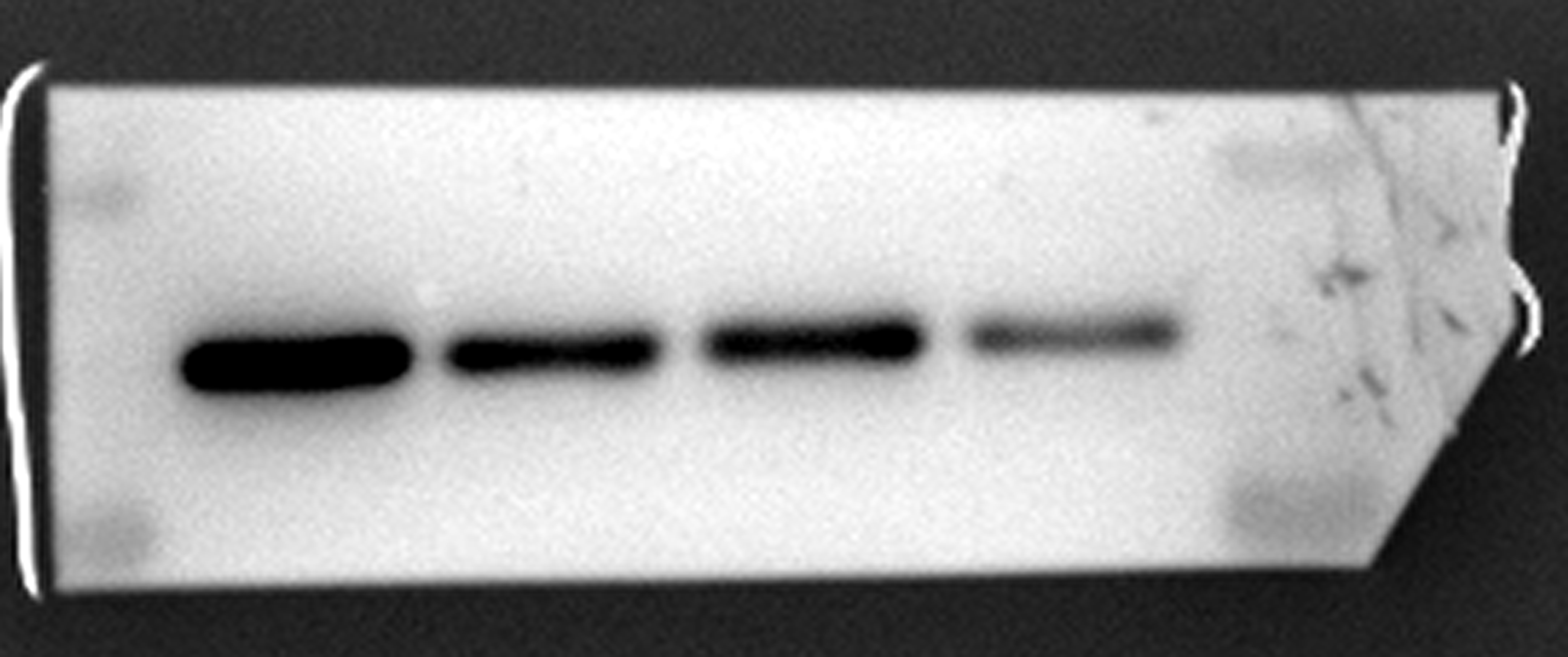

Supplement: Supplemental Material [file KBIE_A_2053804_SM3009.zip › Fig2F_p_ACC.tif]

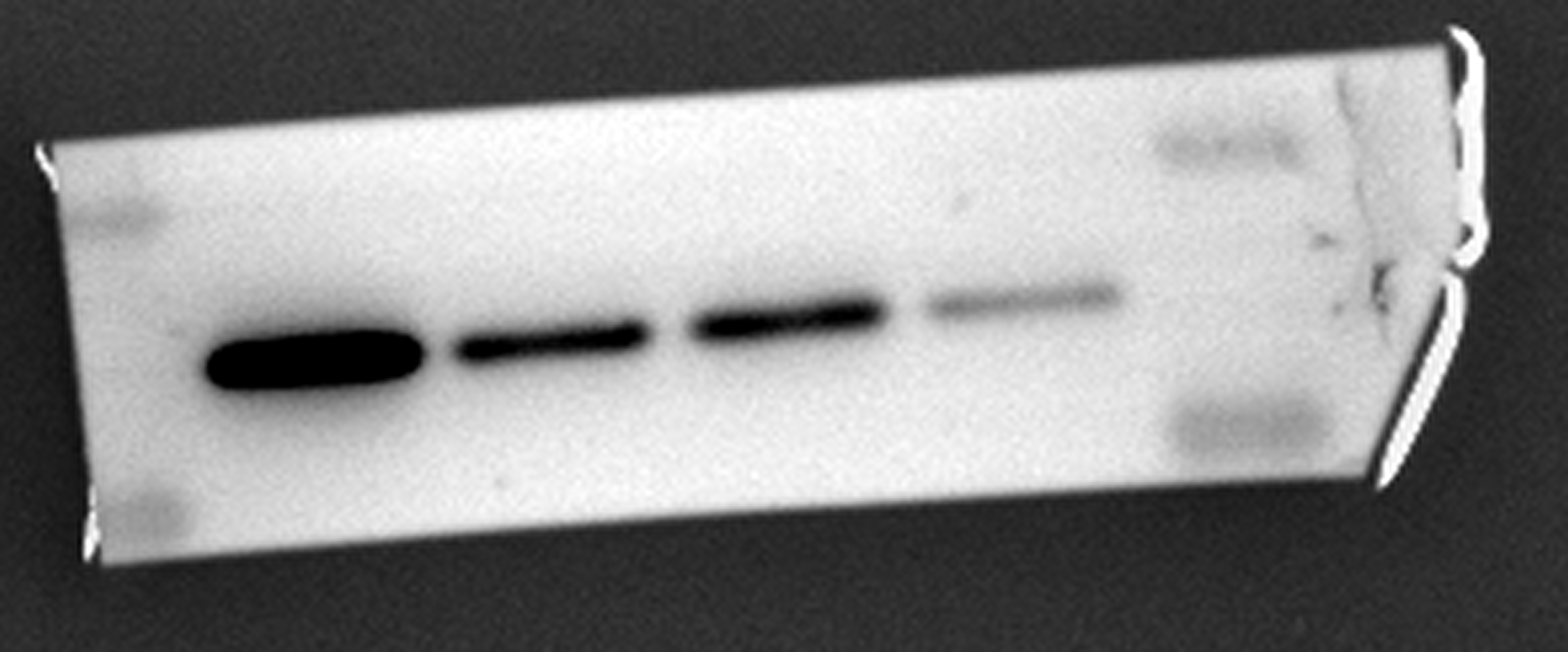

Supplement: Supplemental Material [file KBIE_A_2053804_SM3009.zip › Fig2F_p_AMPK.tif]

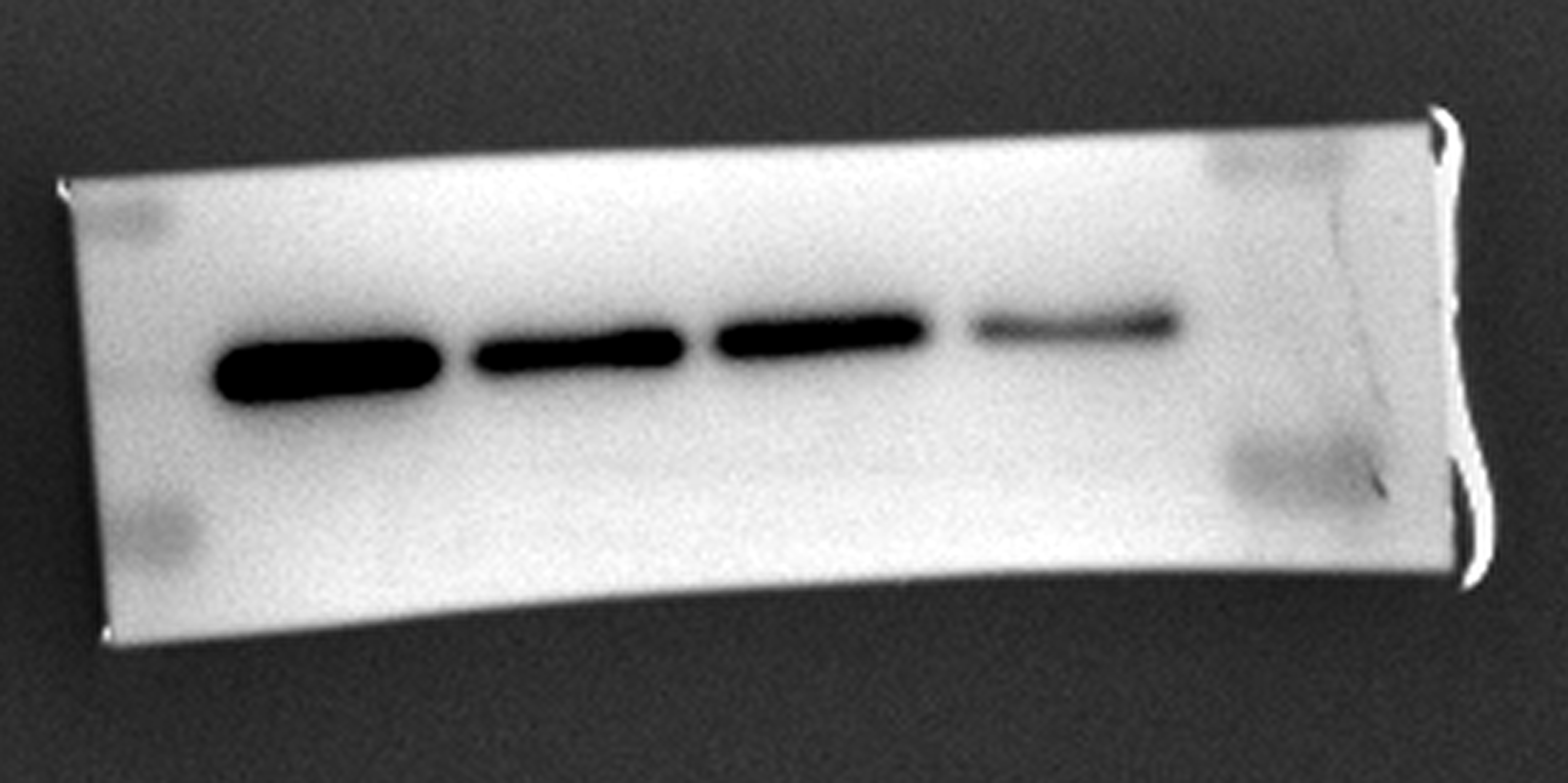

Supplement: Supplemental Material [file KBIE_A_2053804_SM3009.zip › Fig2F_p_LKB1.tif]

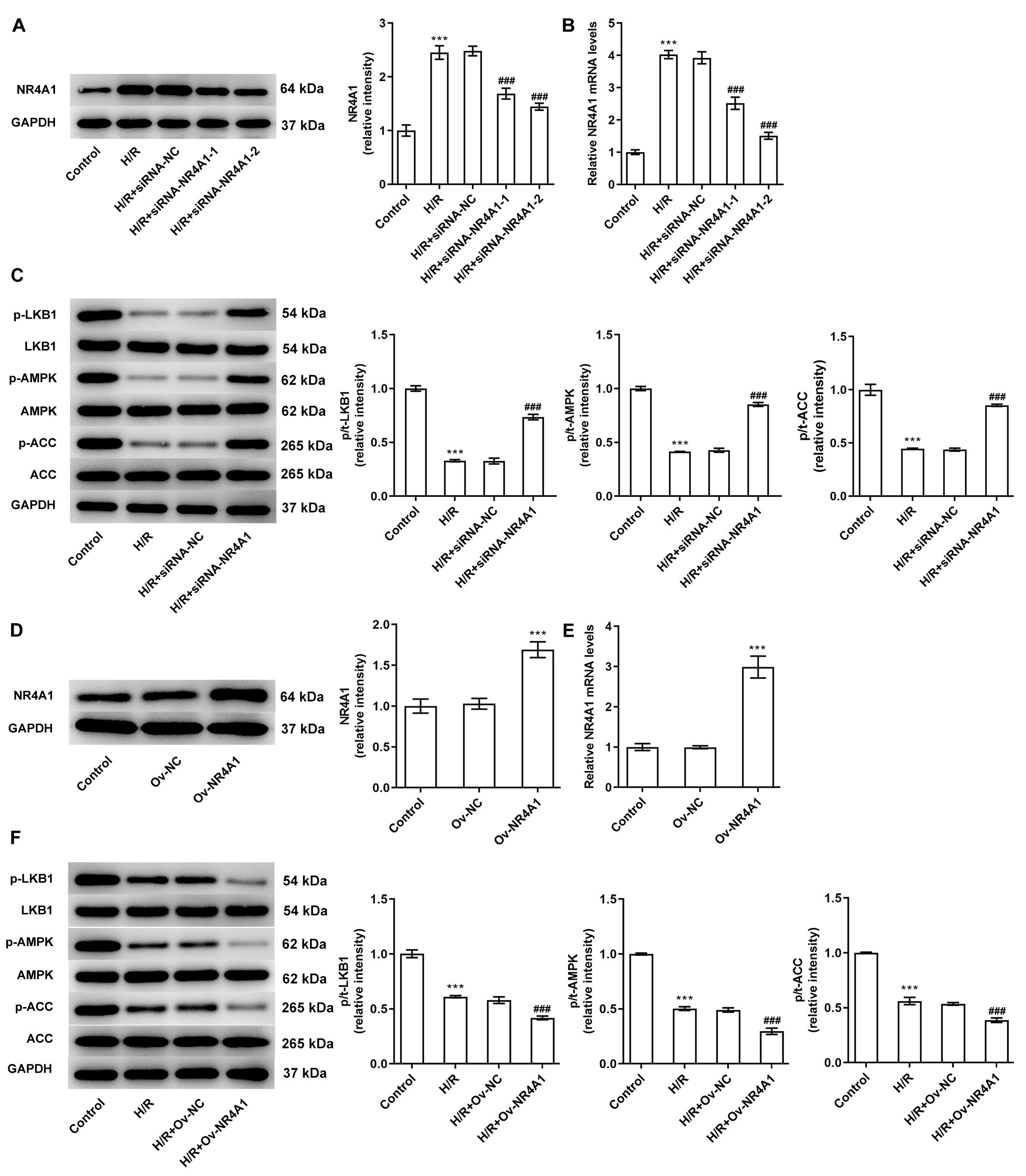

Supplement: Supplemental Material [file KBIE_A_2053804_SM3009.zip › fig2_revised.tif]

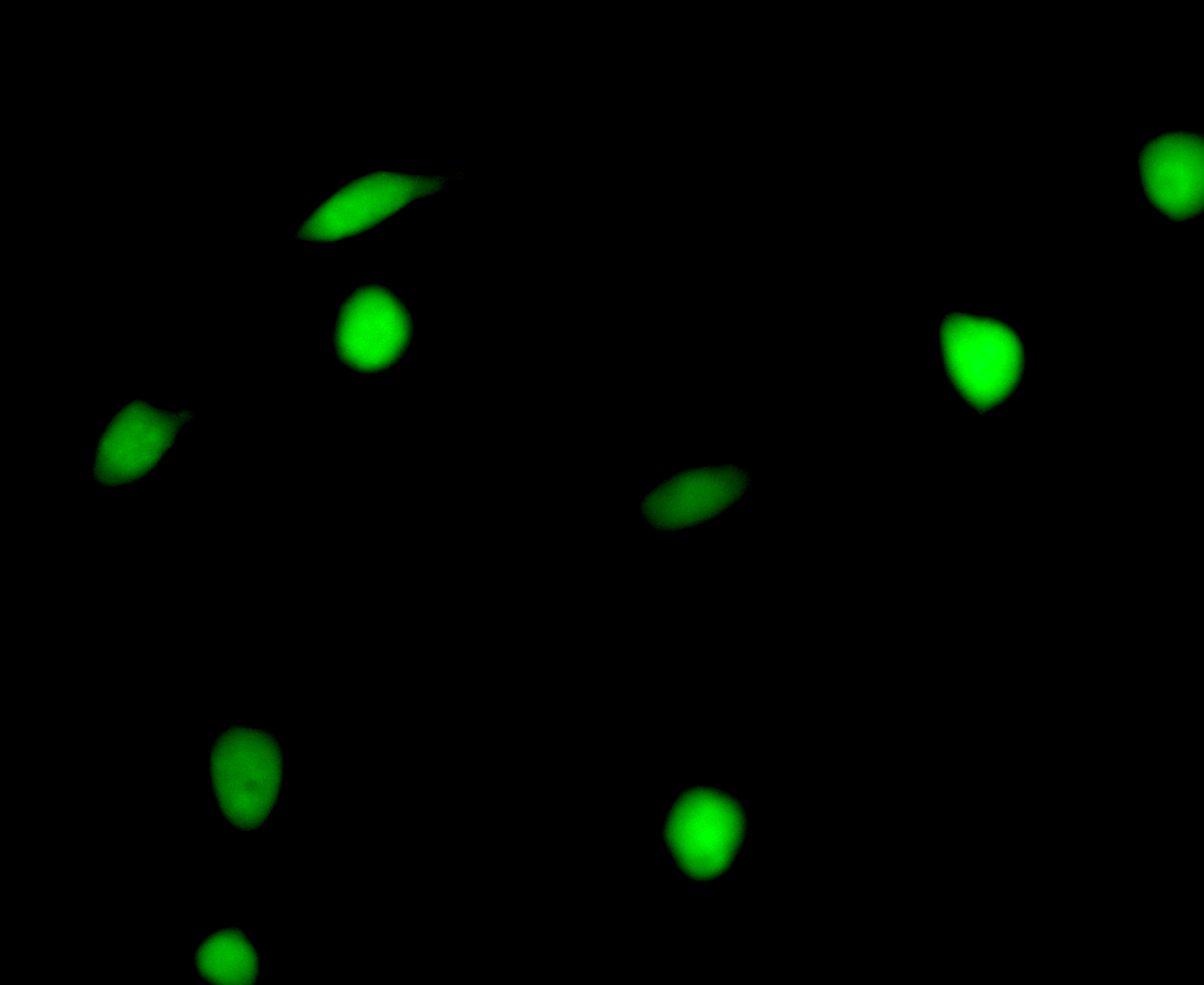

Supplement: Supplemental Material [file KBIE_A_2053804_SM3009.zip › Fig3B_Control.tif]

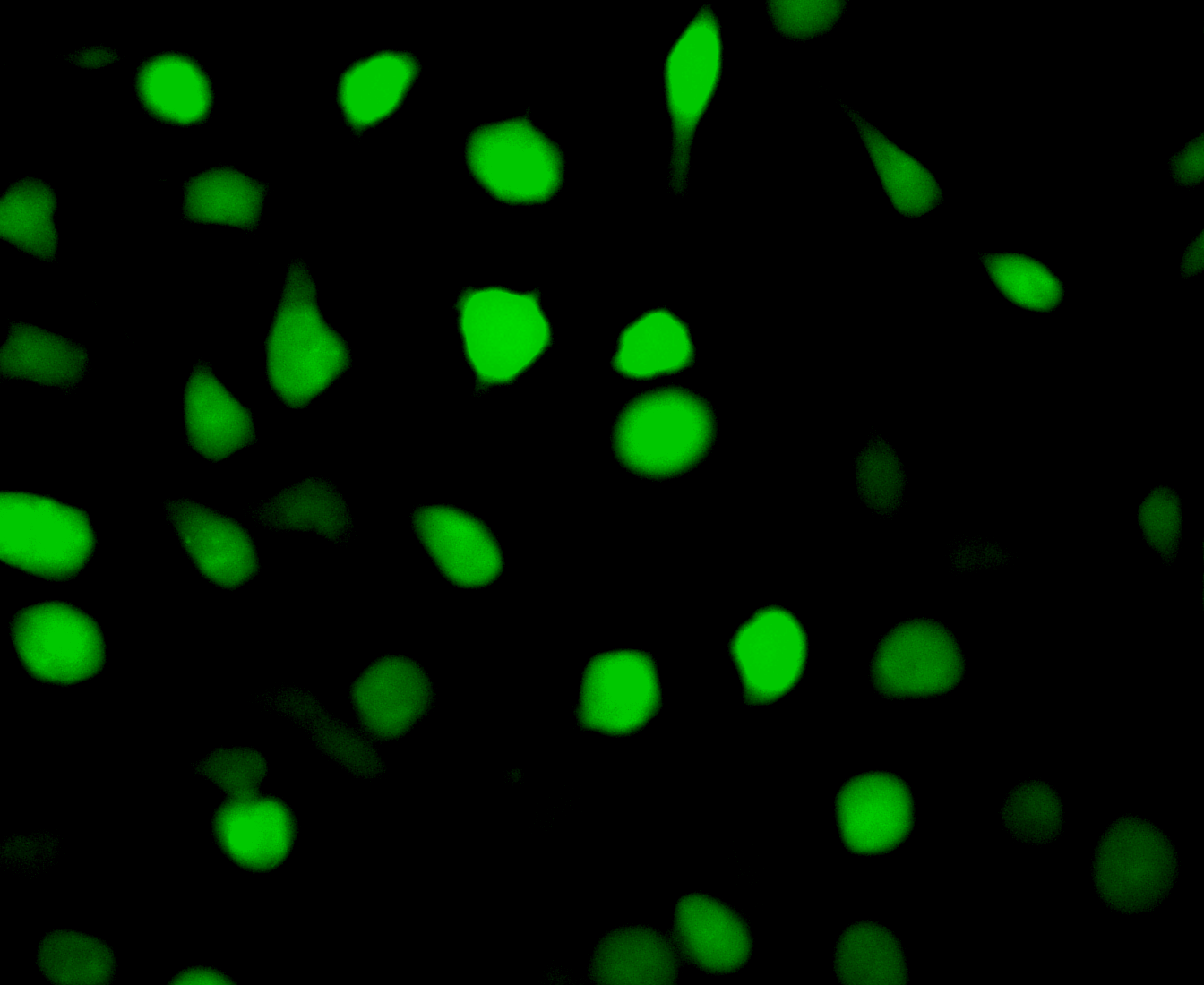

Supplement: Supplemental Material [file KBIE_A_2053804_SM3009.zip › Fig3B_HR.tif]

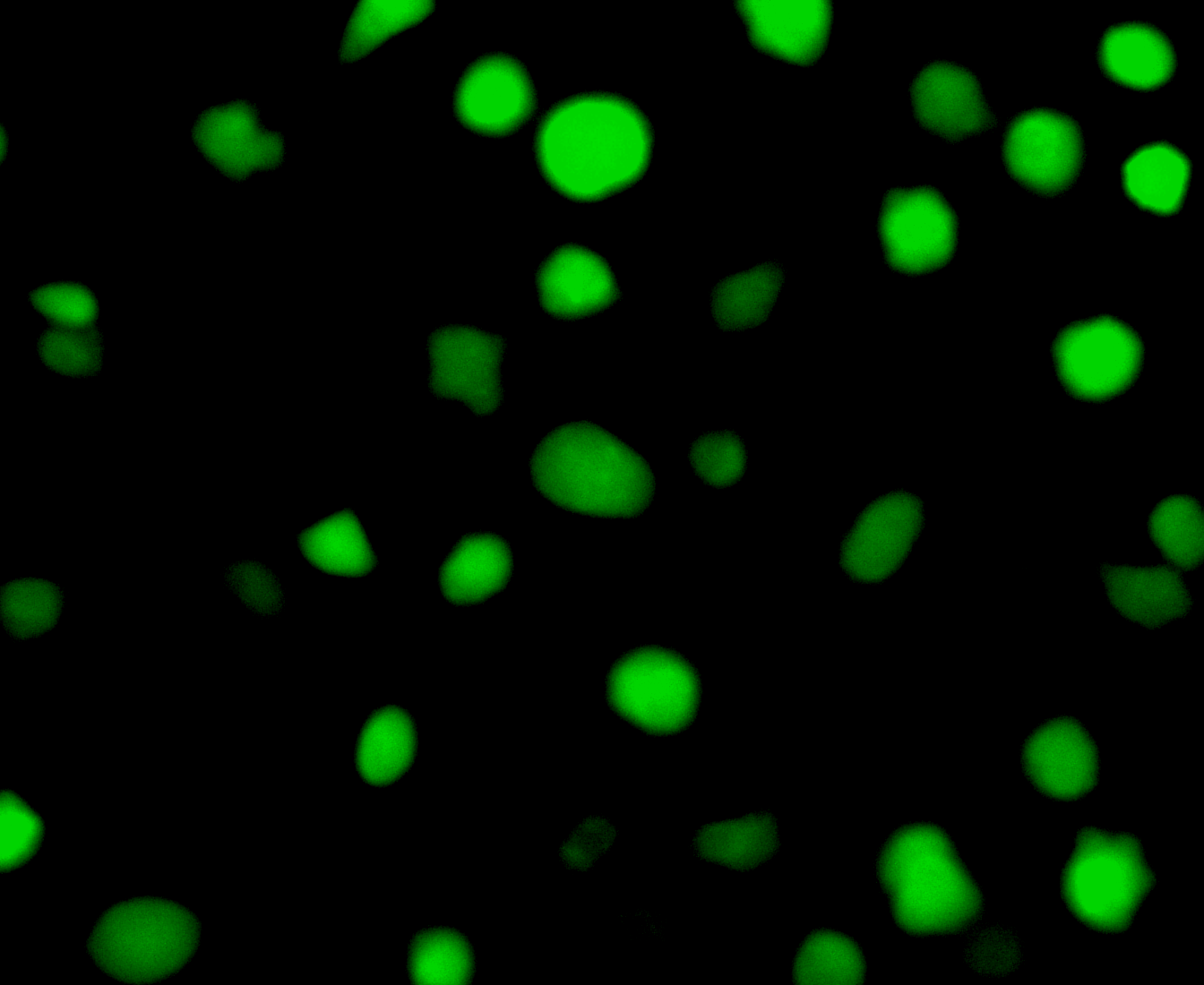

Supplement: Supplemental Material [file KBIE_A_2053804_SM3009.zip › Fig3B_HR_siRNA_NC.tif]

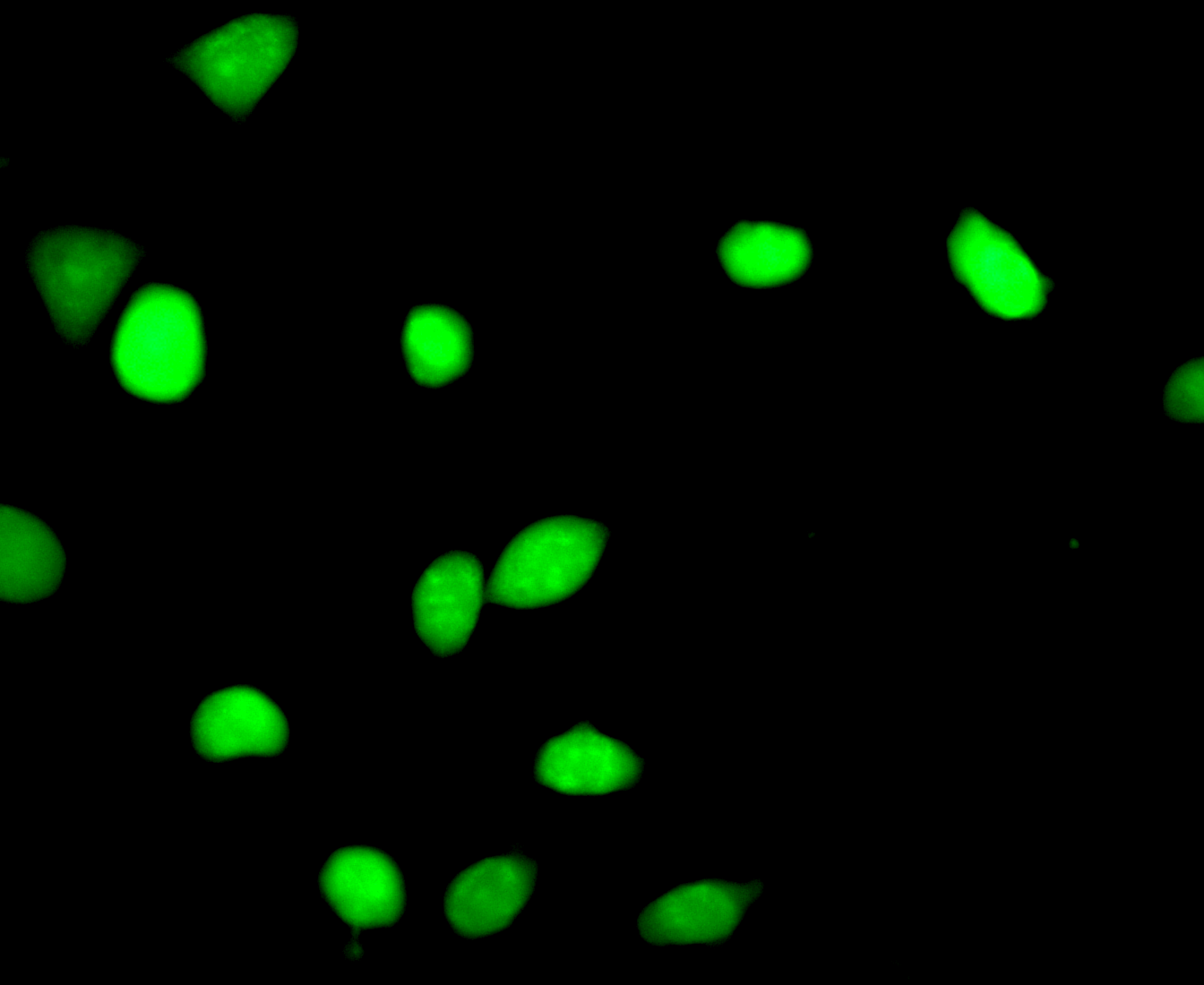

Supplement: Supplemental Material [file KBIE_A_2053804_SM3009.zip › Fig3B_HR_siRNA_NR4A1.tif]

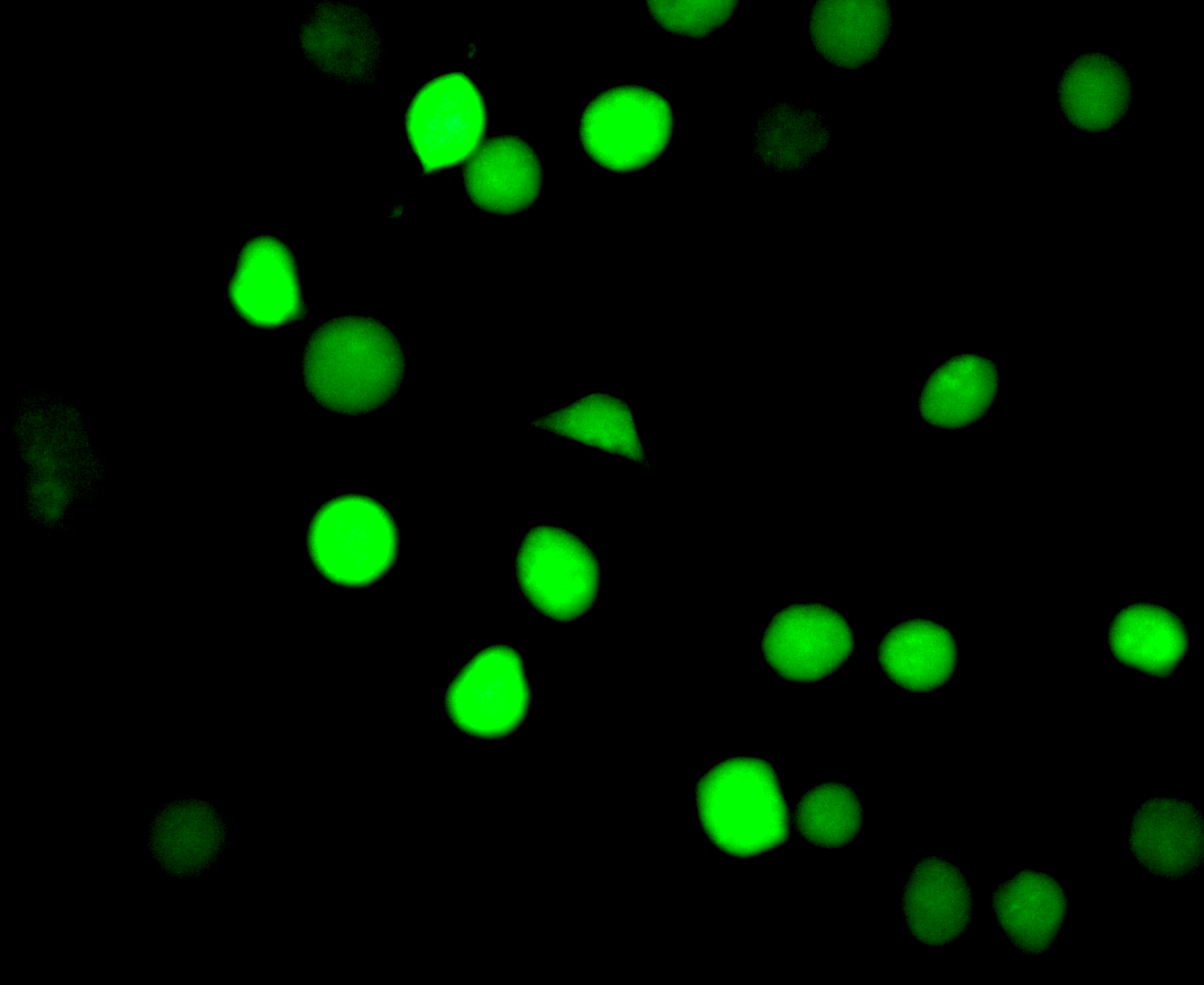

Supplement: Supplemental Material [file KBIE_A_2053804_SM3009.zip › Fig3B_HR_siRNA_NR4A1_radicicol.tif]

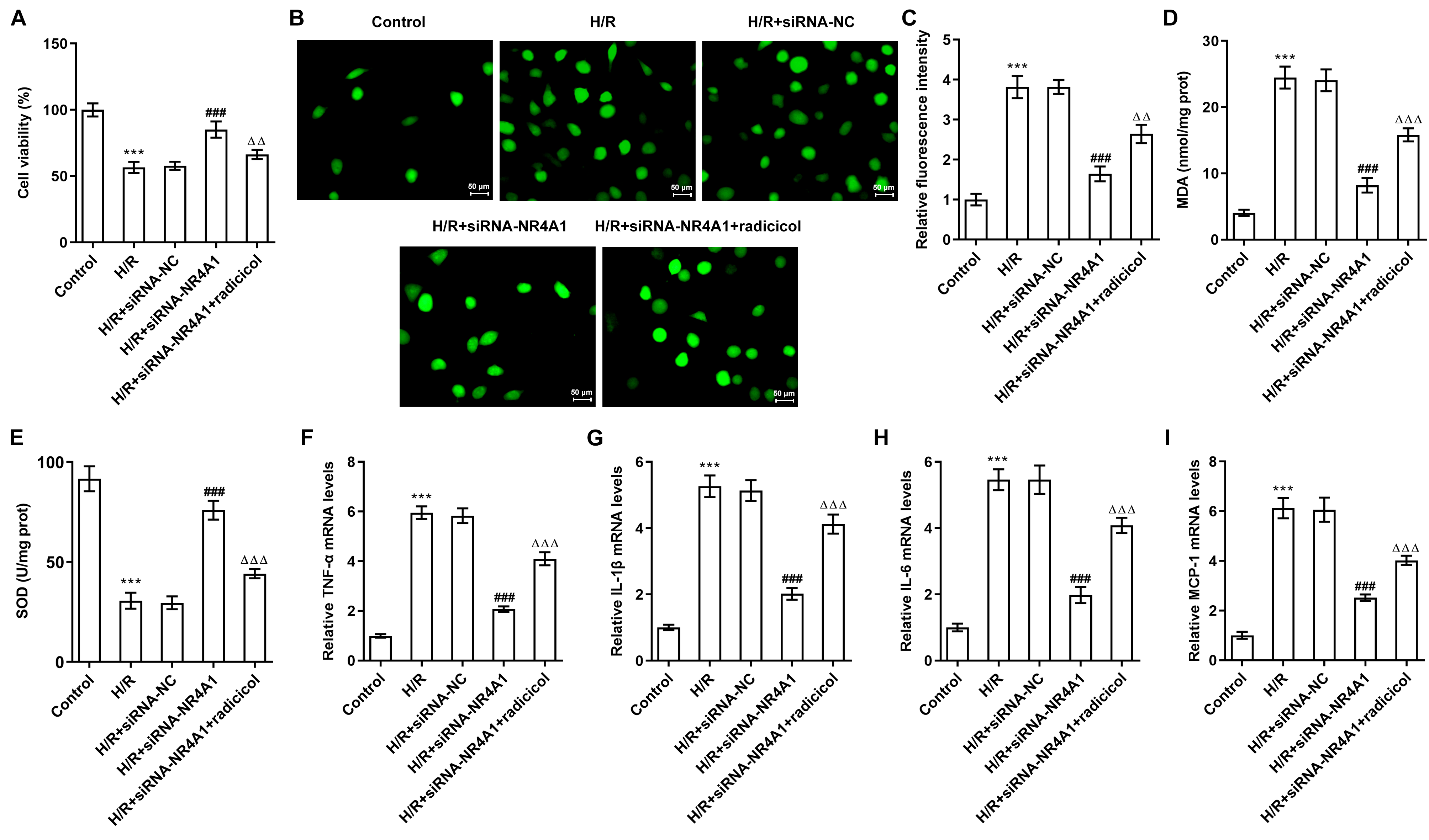

Supplement: Supplemental Material [file KBIE_A_2053804_SM3009.zip › fig3_revised.tif]

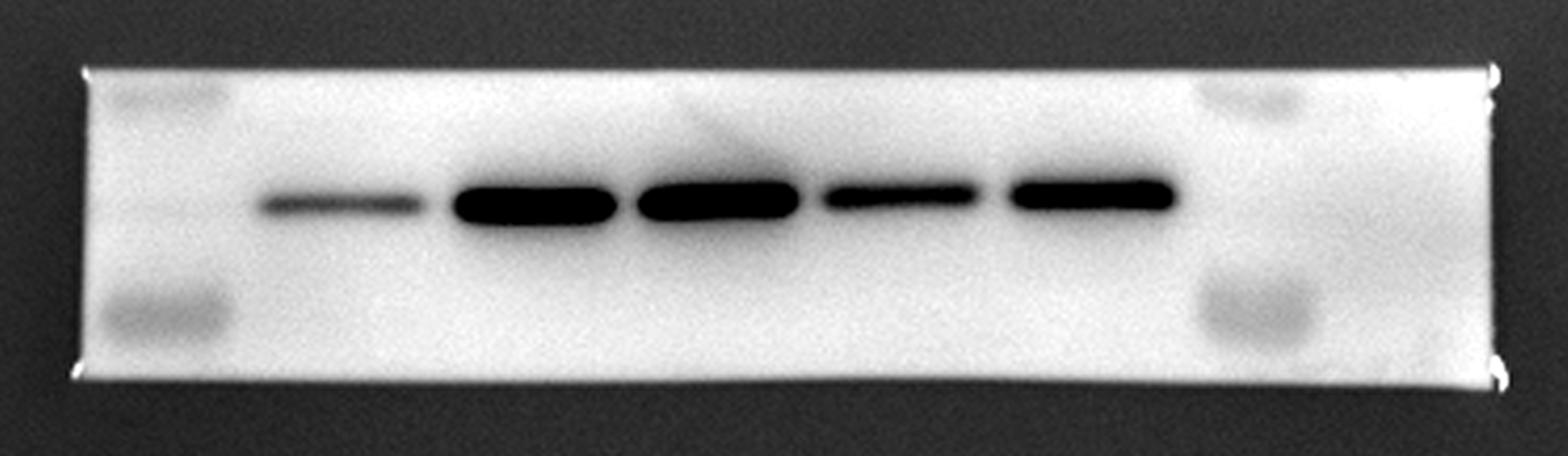

Supplement: Supplemental Material [file KBIE_A_2053804_SM3009.zip › Fig4C_Bax.tif]

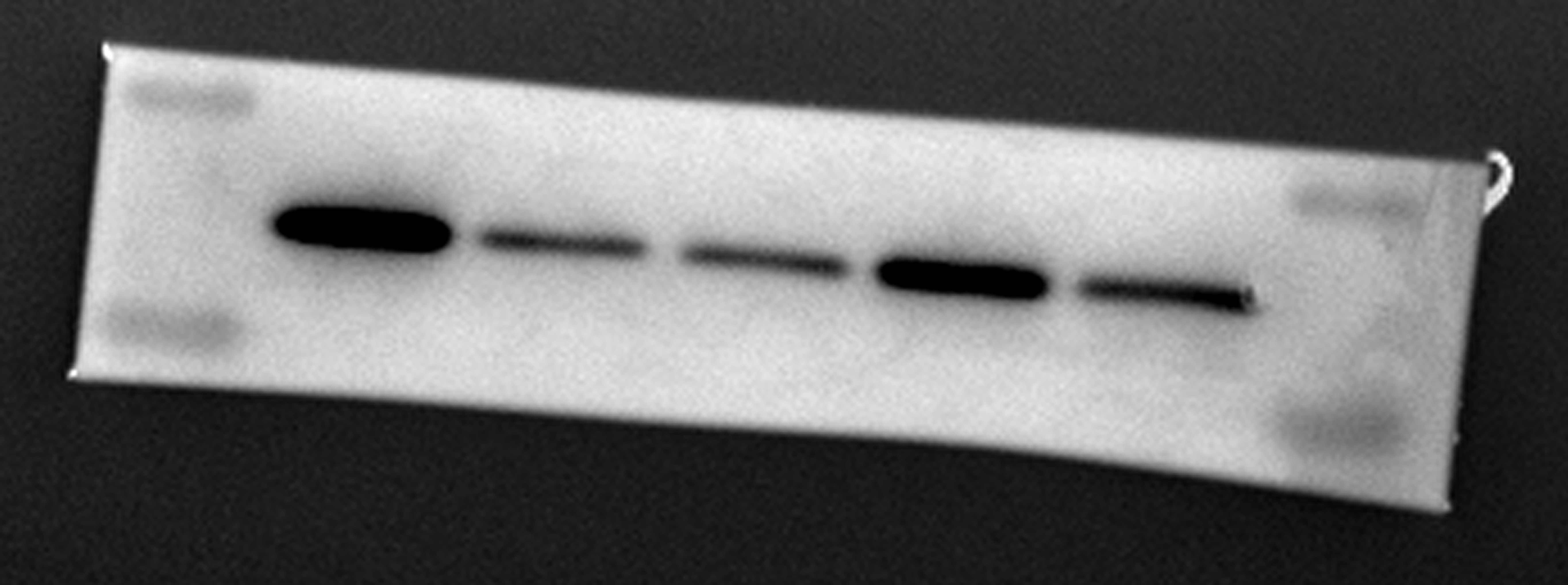

Supplement: Supplemental Material [file KBIE_A_2053804_SM3009.zip › Fig4C_Bcl_2.tif]

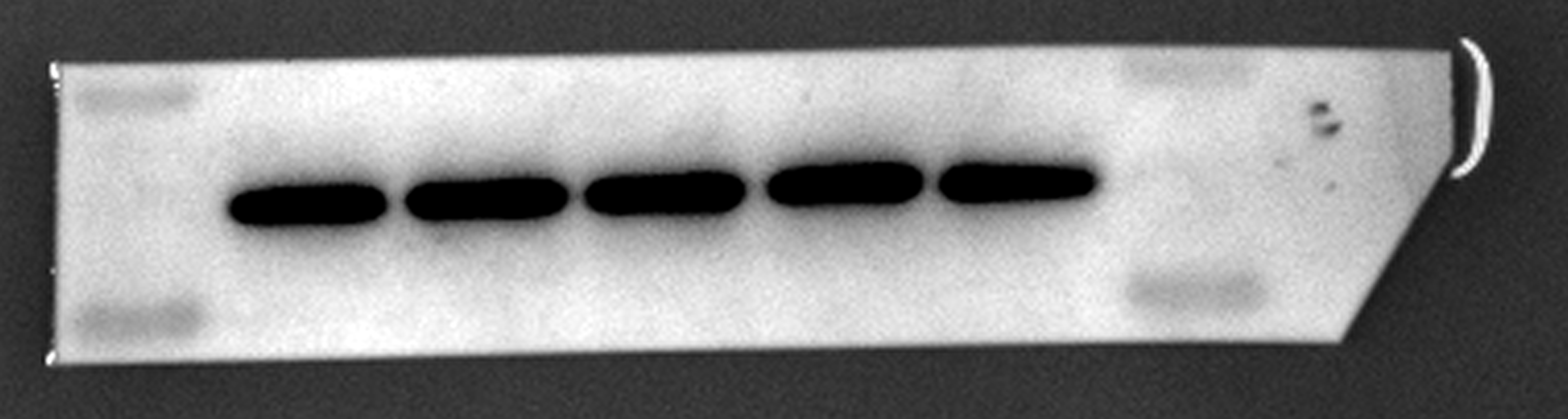

Supplement: Supplemental Material [file KBIE_A_2053804_SM3009.zip › Fig4C_caspase3.tif]

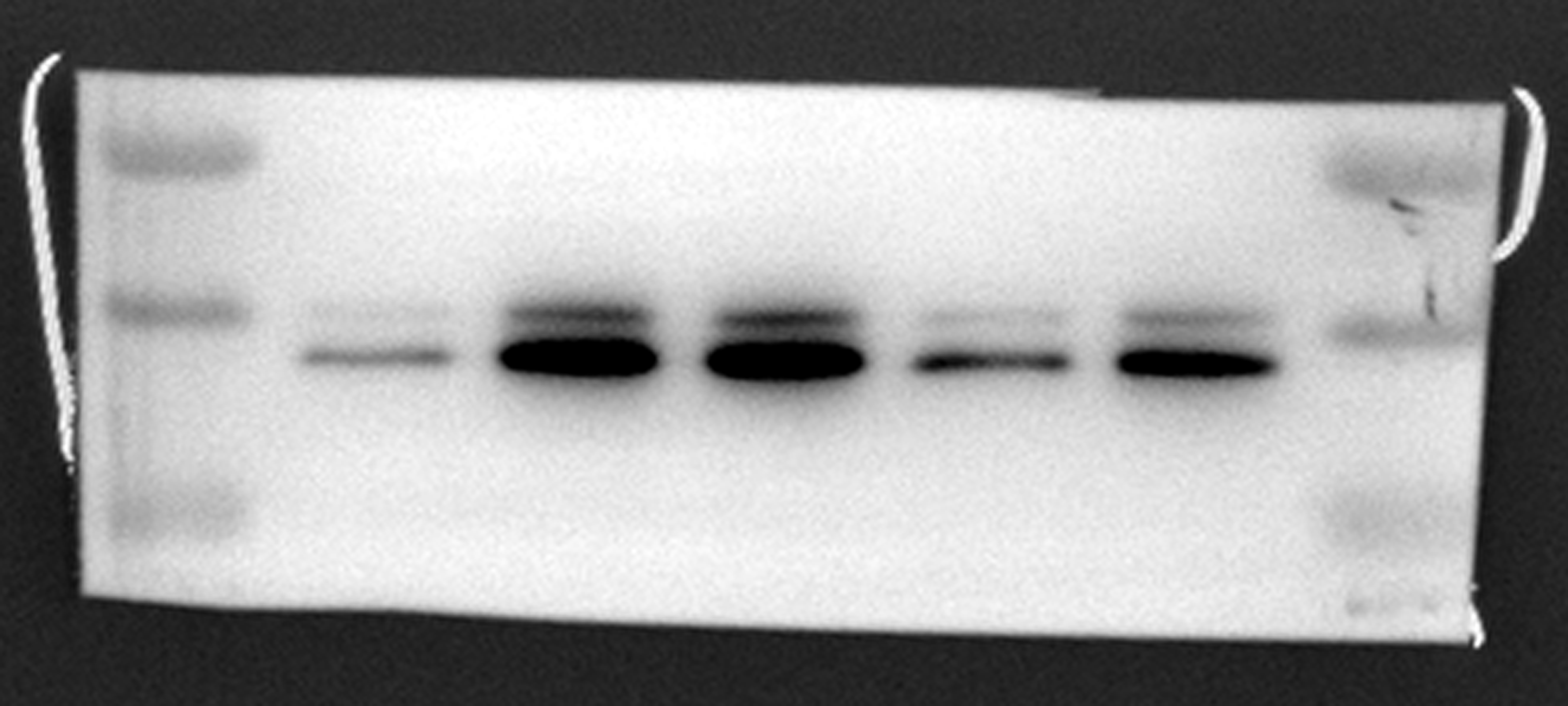

Supplement: Supplemental Material [file KBIE_A_2053804_SM3009.zip › Fig4C_cleaved caspase3.tif]

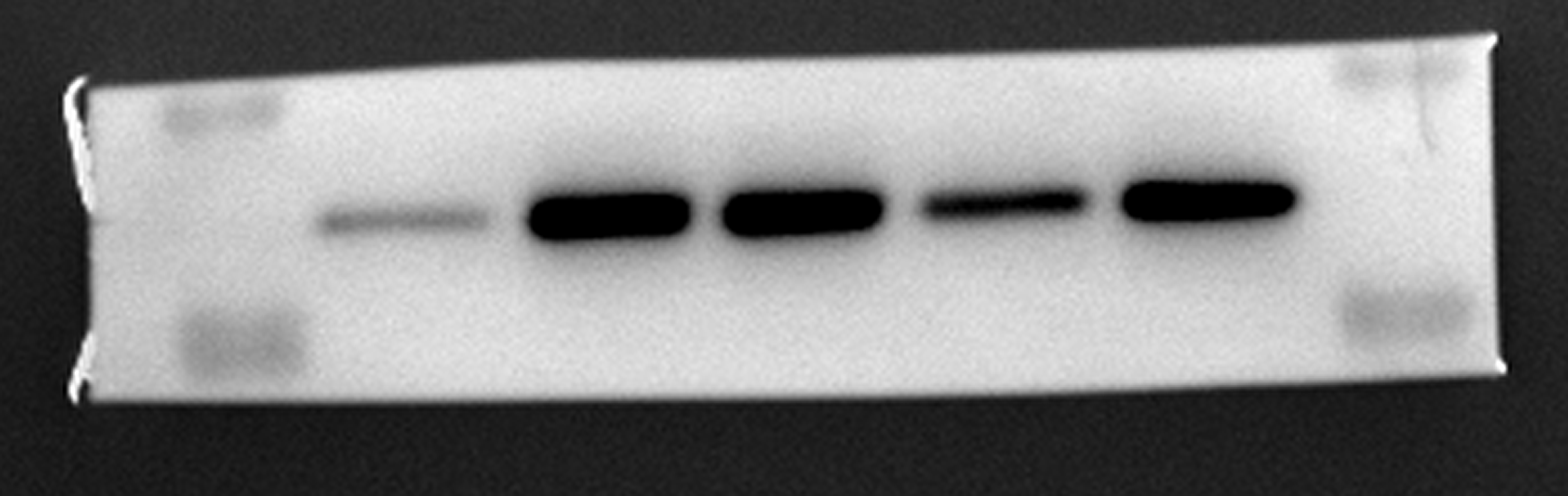

Supplement: Supplemental Material [file KBIE_A_2053804_SM3009.zip › Fig4C_cleaved PARP.tif]

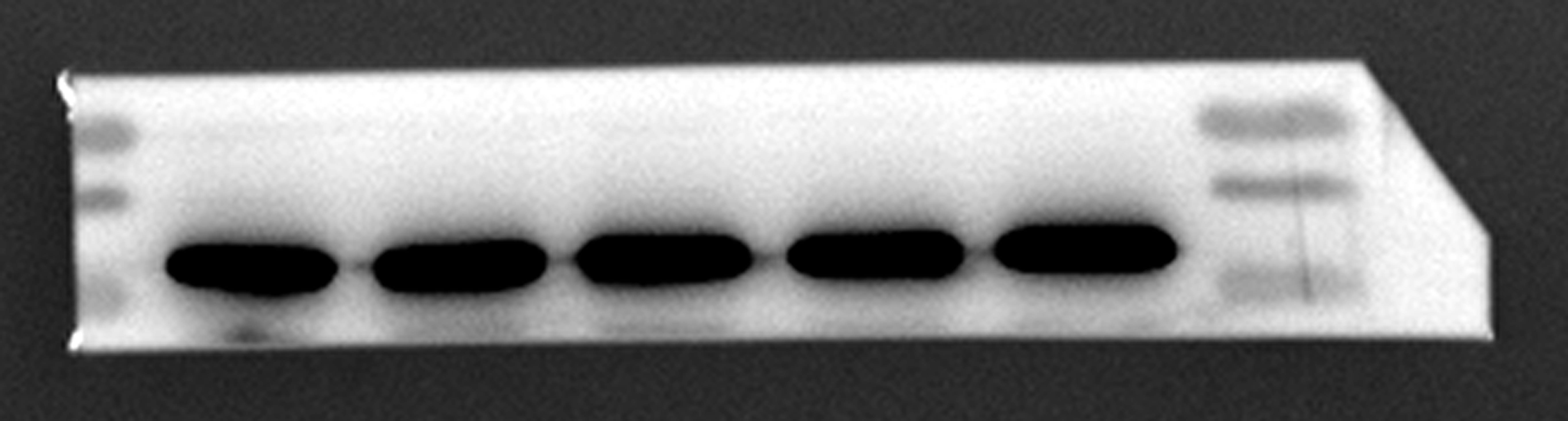

Supplement: Supplemental Material [file KBIE_A_2053804_SM3009.zip › Fig4C_GAPDH.tif]

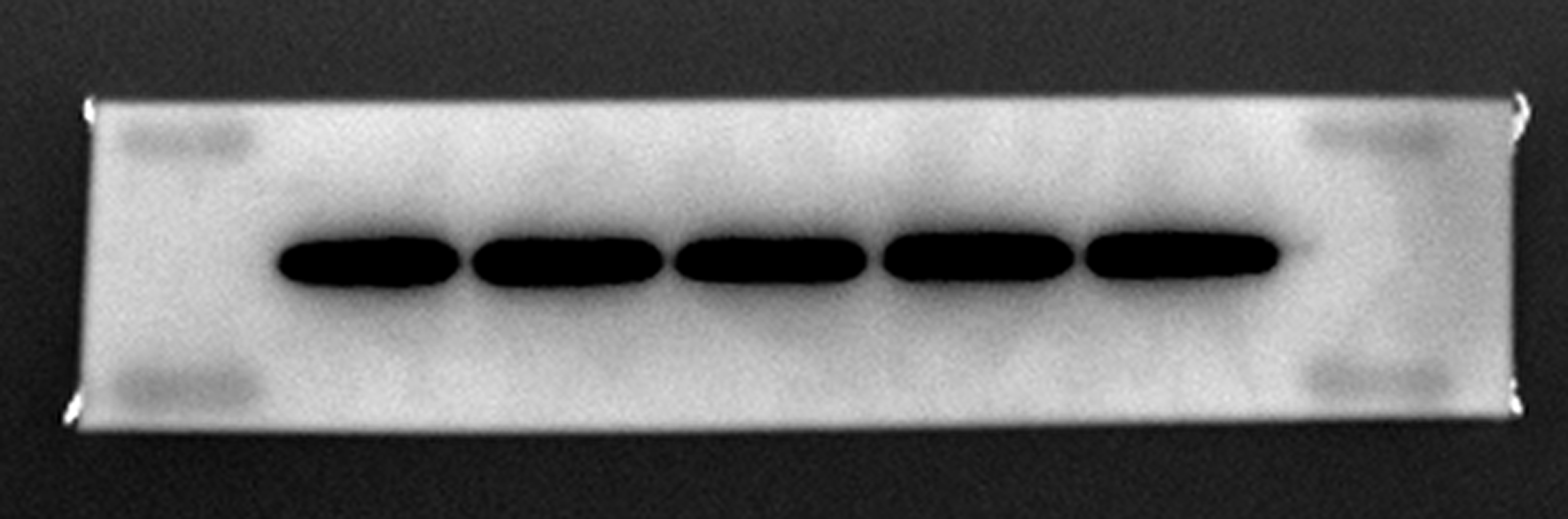

Supplement: Supplemental Material [file KBIE_A_2053804_SM3009.zip › Fig4C_PARP.tif]

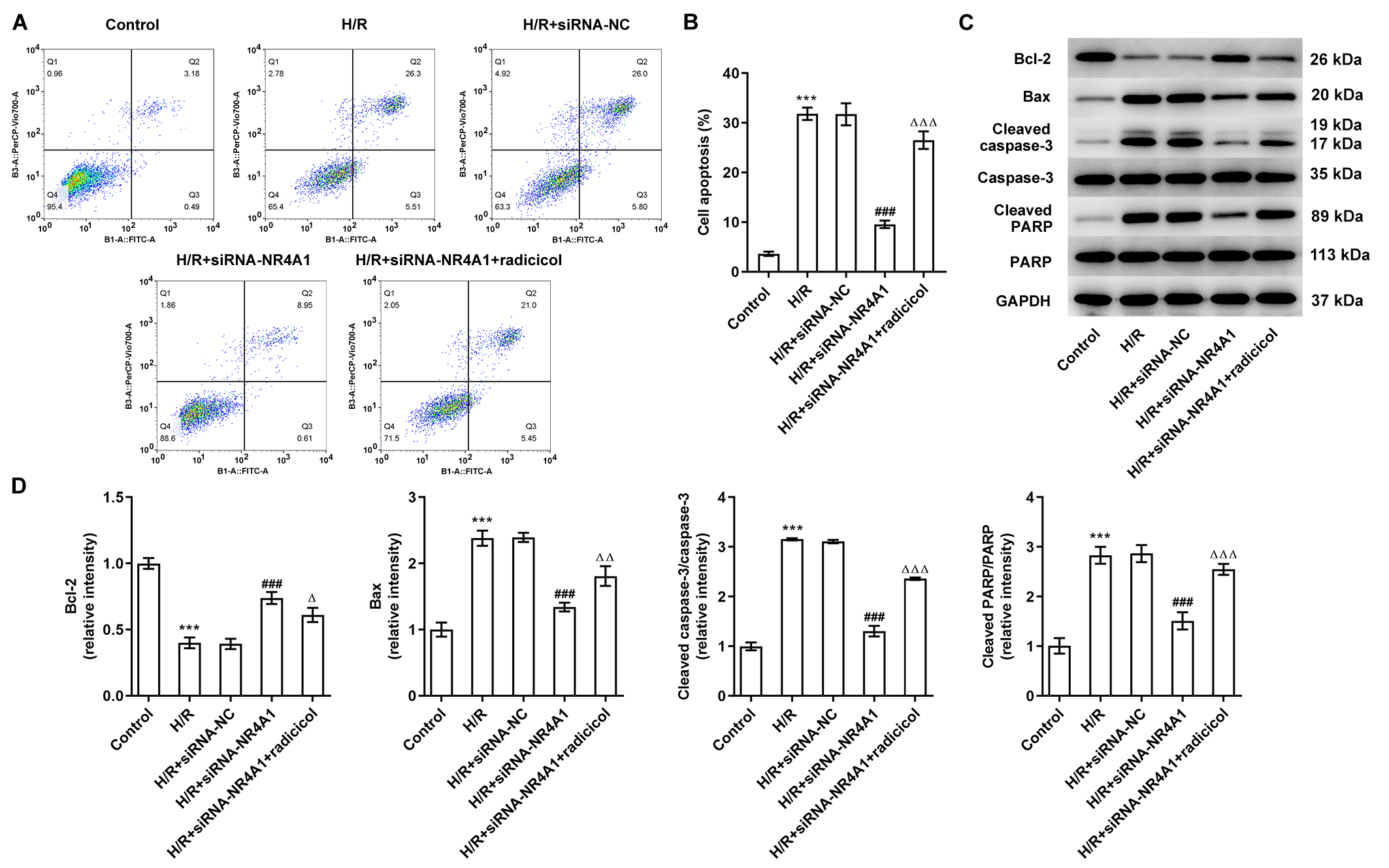

Supplement: Supplemental Material [file KBIE_A_2053804_SM3009.zip › fig4_revised.tif]

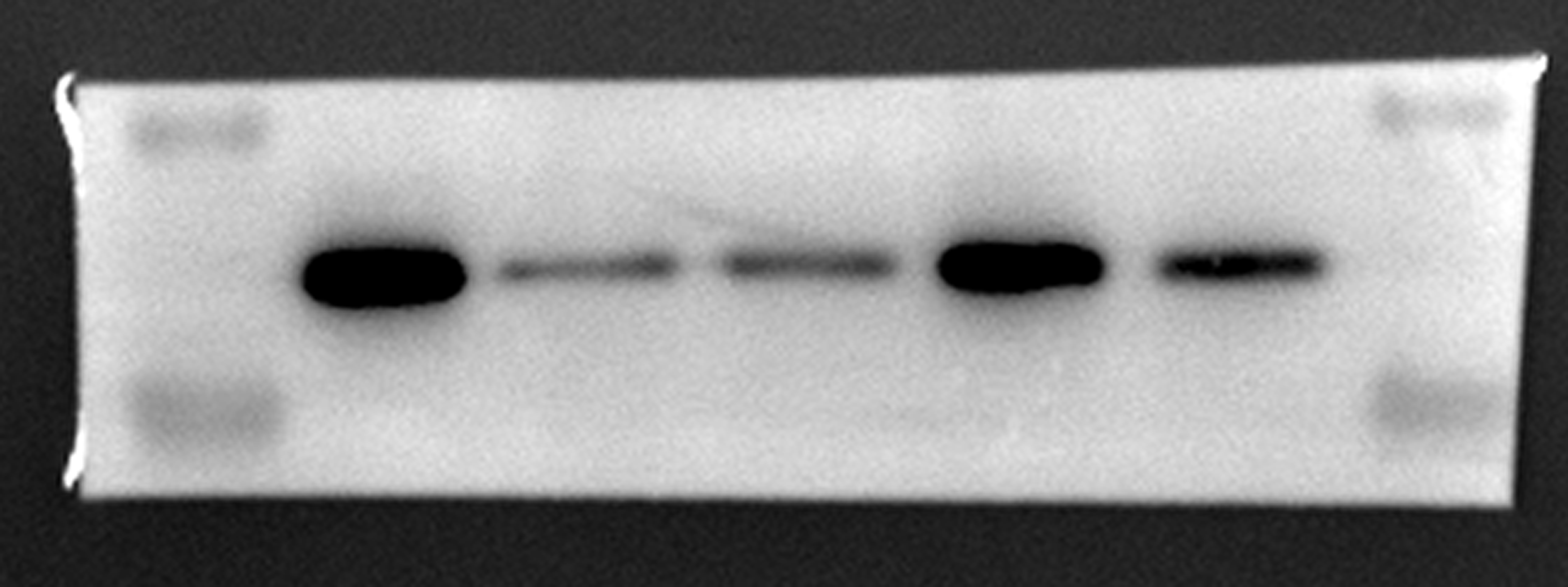

Supplement: Supplemental Material [file KBIE_A_2053804_SM3009.zip › Fig5_ATG5.tif]

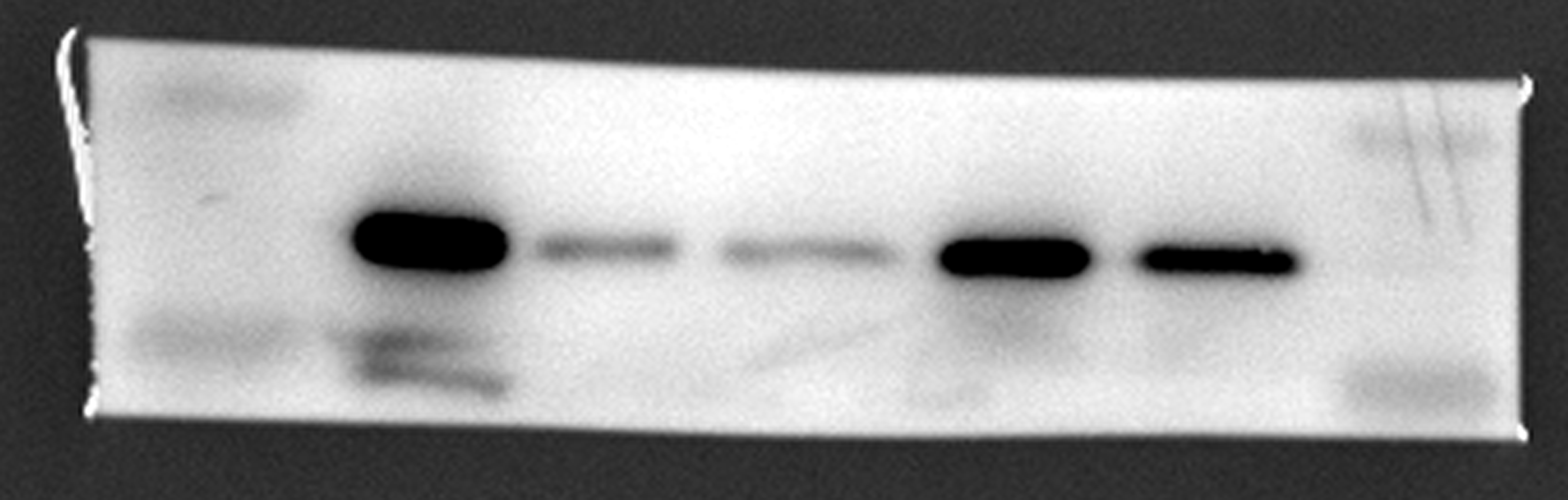

Supplement: Supplemental Material [file KBIE_A_2053804_SM3009.zip › Fig5_ATG7.tif]

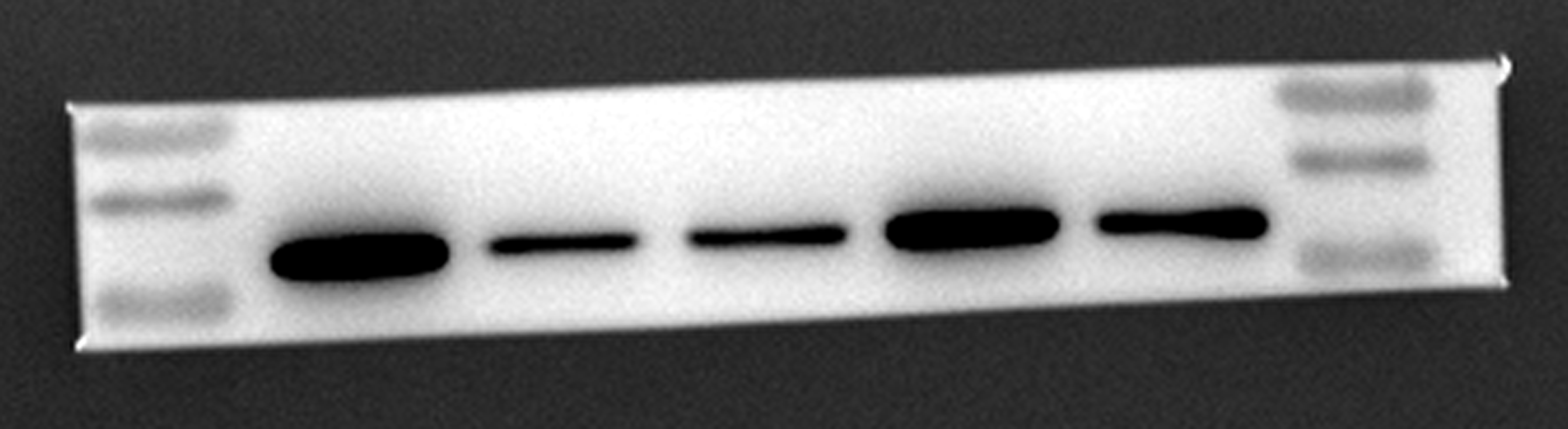

Supplement: Supplemental Material [file KBIE_A_2053804_SM3009.zip › Fig5_Beclin1.tif]

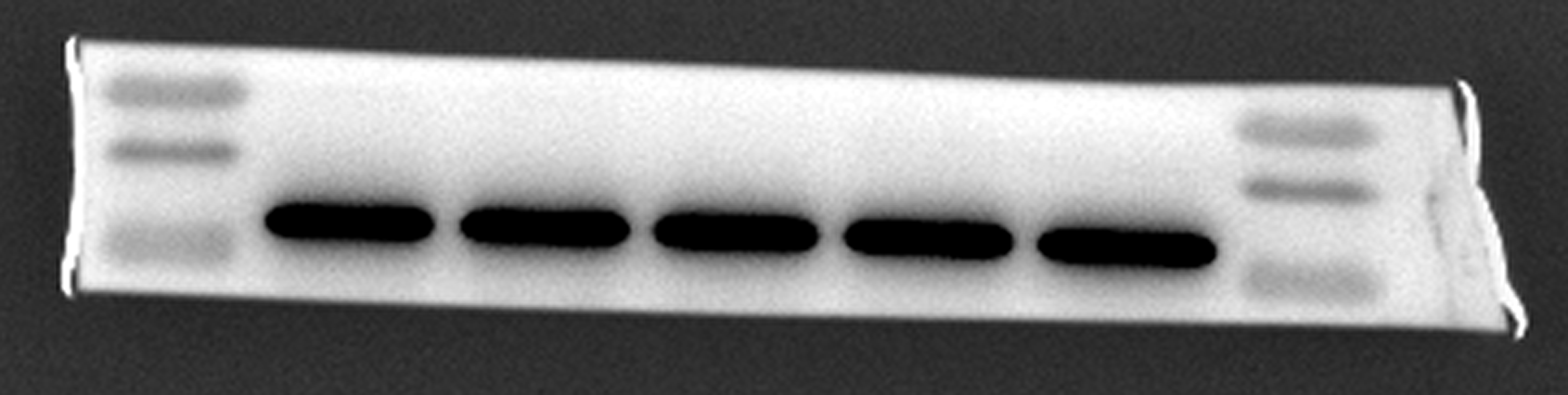

Supplement: Supplemental Material [file KBIE_A_2053804_SM3009.zip › Fig5_GAPDH.tif]

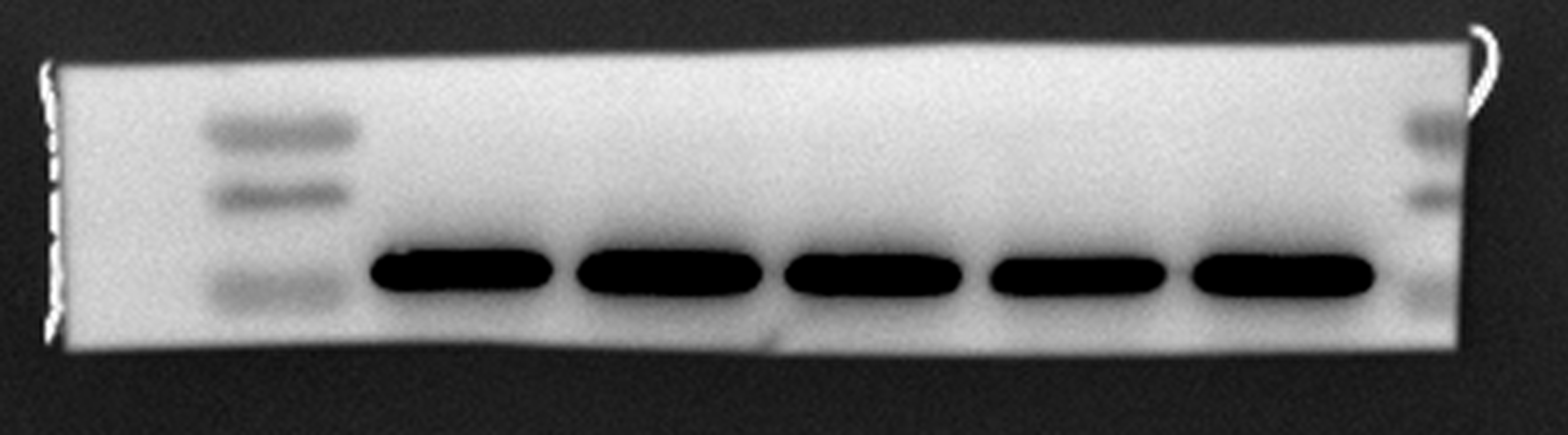

Supplement: Supplemental Material [file KBIE_A_2053804_SM3009.zip › Fig5_GAPDH_1.tif]

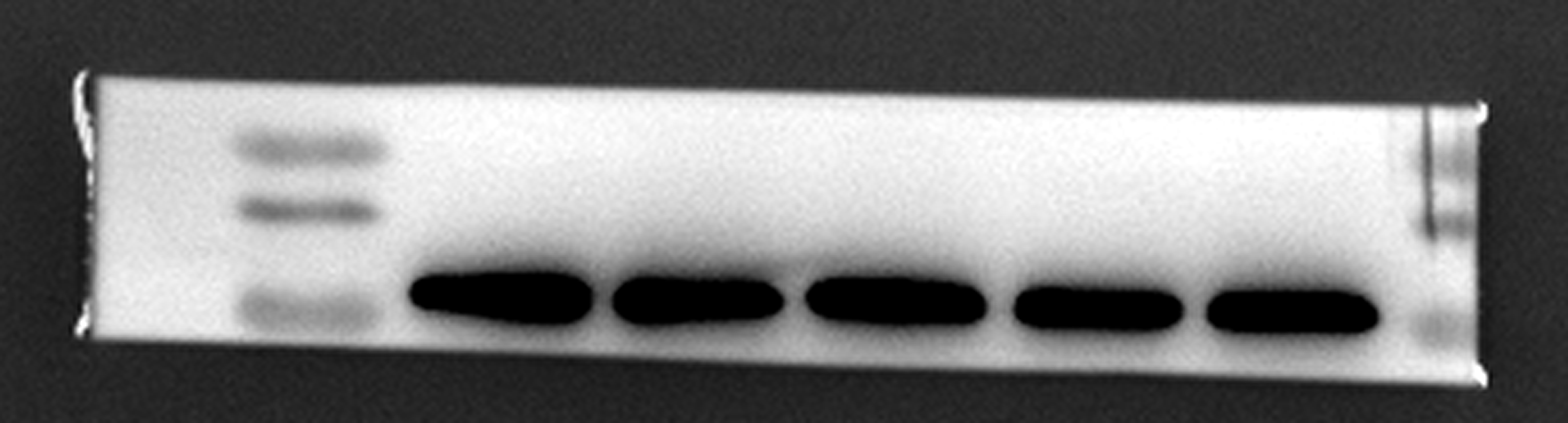

Supplement: Supplemental Material [file KBIE_A_2053804_SM3009.zip › Fig5_GAPDH_2.tif]

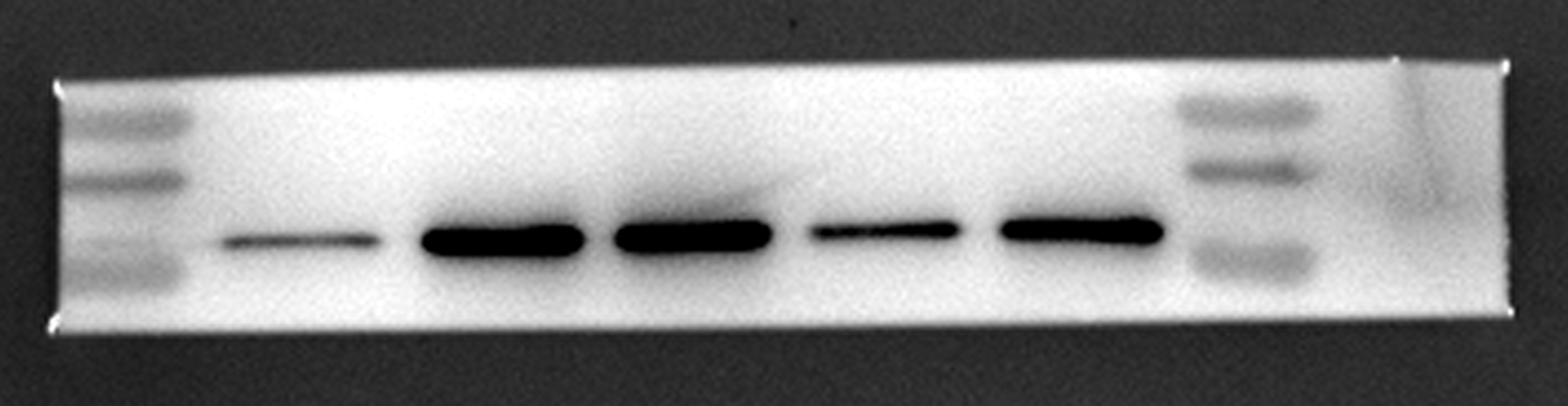

Supplement: Supplemental Material [file KBIE_A_2053804_SM3009.zip › Fig5_p62.tif]

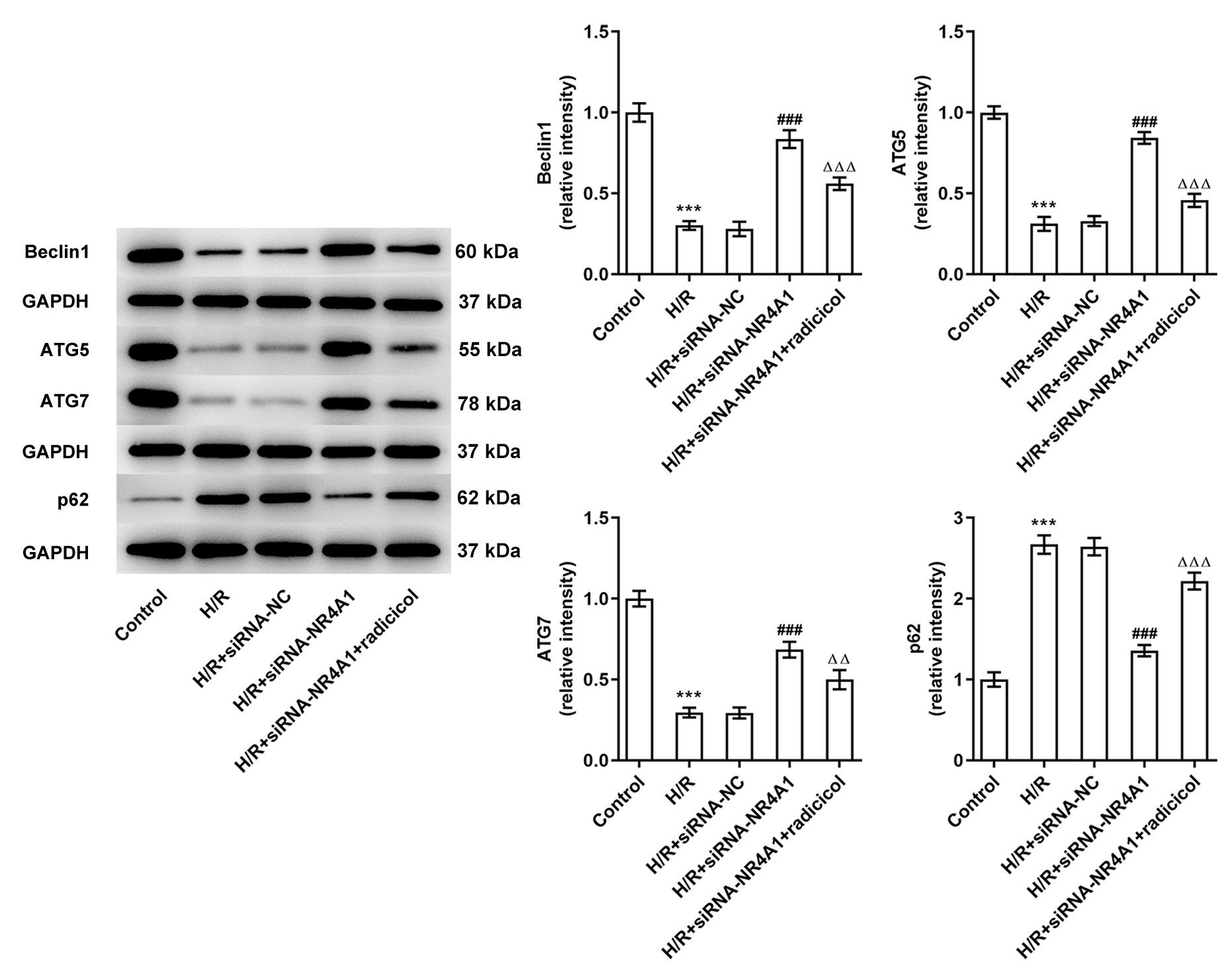

Supplement: Supplemental Material [file KBIE_A_2053804_SM3009.zip › fig5_revised.tif]
